# New evidence: Metformin unsuitable as routine adjuvant for breast cancer: a drug-target mendelian randomization analysis

**DOI:** 10.1186/s12885-024-12453-w

**Published:** 2024-06-06

**Authors:** Jing-Xuan Xu, Qi-Long Zhu, Yu-Miao Bi, Yu-Chong Peng

**Affiliations:** 1 Department of General Surgery, Chongqing Hospital of Traditional Chinese Medicine, Chongqing, 400021 China; 2 Department of Hepatobiliary Surgery, Guangxi Medical University Cancer Hospital, Nanning, Guangxi Province 530021 China; 3 Pharmacy Department, The Ninth People’s Hospital of Chongqing, Chongqing, 400015 China

**Keywords:** Breast cancer, Metformin, Causal relationship, Drug-target mendelian randomization

## Abstract

**Purpose:**

The potential efficacy of metformin in breast cancer (BC) has been hotly discussed but never conclusive. This genetics-based study aimed to evaluate the relationships between metformin targets and BC risk.

**Methods:**

Metformin targets from DrugBank and genome-wide association study (GWAS) data from IEU OpenGWAS and FinnGen were used to investigate the breast cancer (BC)-metformin causal link with various Mendelian Randomization (MR) methods (e.g., inverse-variance-weighting). The genetic association between type 2 diabetes (T2D) and the drug target of metformin was also analyzed as a positive control. Sensitivity and pleiotropic tests ensured reliability.

**Results:**

The primary targets of metformin are PRKAB1, ETFDH and GPD1L. We found a causal association between PRKAB1 and T2D (odds ratio [OR] 0.959, *P* = 0.002), but no causal relationship was observed between metformin targets and overall BC risk (PRKAB1: OR 0.990, *P* = 0.530; ETFDH: OR 0.986, *P* = 0.592; GPD1L: OR 1.002, *P* = 0.806). A noteworthy causal relationship was observed between ETFDH and estrogen receptor (ER)-positive BC (OR 0.867, *P* = 0.018), and between GPD1L and human epidermal growth factor receptor 2 (HER2)-negative BC (OR 0.966, *P* = 0.040). Other group analyses did not yield positive results.

**Conclusion:**

The star target of metformin, PRKAB1, does not exhibit a substantial causal association with the risk of BC. Conversely, metformin, acting as an inhibitor of ETFDH and GPD1L, may potentially elevate the likelihood of developing ER-positive BC and HER2-negative BC. Consequently, it is not advisable to employ metformin as a standard supplementary therapy for BC patients without T2D.

**Supplementary Information:**

The online version contains supplementary material available at 10.1186/s12885-024-12453-w.

## Introduction

Breast cancer (BC) is the most prevalent malignant tumor among women, exhibiting an escalating incidence globally and serving as a leading cause of cancer-related mortality in women worldwide [1, 2]. Concurrently, Type 2 diabetes (T2D) has emerged as a significant public health issue on a global scale [3]. Research investigations have demonstrated a positive association between T2D and an elevated risk of BC [4], potentially attributable to the activation of insulin or insulin-like growth factor receptors within breast epithelial tissue, or the alteration of sex hormone levels due to insulin resistance and hyperinsulinemia [5]. In light of these mechanisms, metformin, a well-established therapeutic approach for T2D, is believed to have the potential to mitigate the risk of breast cancer and enhance BC outcomes [6]. Additionally, metformin exerts its effects on the AMPK and mTOR pathways, thereby potentially impeding the growth of BC [7, 8]. Although preclinical investigations offer evidence of metformin’s impact on all subtypes of BC, the translation of these findings to the clinical domain is not without challenges, primarily due to the utilization of supra-physiological concentrations of glucose, insulin, and metformin in in vitro and in vivo laboratory models employed in preclinical studies [9, 10].

However, numerous studies have cast doubt on the correlation between T2D and the risk of BC, concurrently underscoring the inadequacy of evidence supporting a definitive impact of metformin on BC patients [11–13]. Furthermore, inconsistencies prevail within the outcomes pertaining to distinct subtypes of BC [14, 15]. A recent investigation demonstrated that the inclusion of metformin, as compared to a placebo, alongside conventional BC treatment did not yield a statistically significant enhancement in invasive disease-free survival among individuals with high-risk operable BC and no preexisting diabetes [10].

Hence, the status of metformin as a potential standard adjuvant therapy for breast cancer remains inconclusive. Similar to the randomized controlled trial (RCT) methodology, the MR approach inherently allocates participants into groups through genetic predictions of drug target perturbation, effectively mitigating the influence of environmental factors due to the random assortment of genetic variants during conception [16]. Furthermore, this approach effectively reduces the potential for reverse causality, as the germline genotype remains unaltered by the onset and progression of disease [17, 18]. An extension of the MR paradigm, the drug-target approach, has been applied to clinical trials in order to predict the effectiveness and potential negative consequences of therapeutic interventions [19]. Using genetic variants to surrogate mechanistic impacts of drug targets, this technique enables characterization of protein function and perturbation of drug targets [20, 21]. In this study, we have employed the drug-target MR methodology to simulate prolonged exposure to metformin in European populations, aiming to evaluate its causal effects on BC and present novel genetic evidence in this regard.

## Materials and methods

### Study overview and data sources

**Fig. 1:**
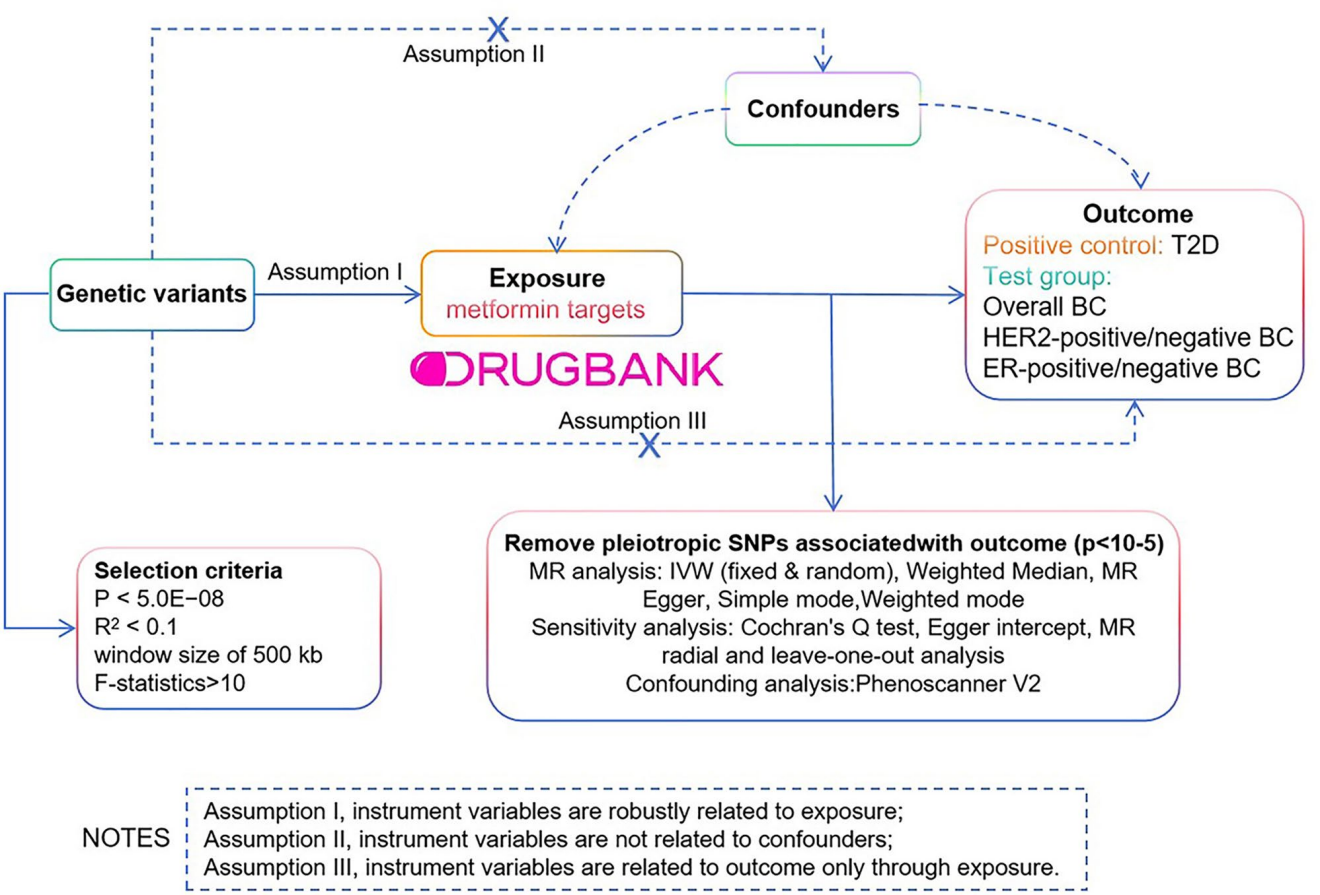
This Figure illustrates a diagram of the MR study. The study’s flowchart is structured upon three core assumptions

We employed a multi-group, two-sample MR design to investigate the potential efficacy of repurposing metformin for BC. Summary statistics of instrument exposure and instrument-outcome associations were derived from large-scale genome-wide association studies (GWAS) conducted in European populations (Fig. 1).

Initially, based on the Drugbank database, the target proteins of metformin were identified [22]. Subsequently, GWAS data corresponding to these genes were obtained from the IEU OpenGWAS project database, and independent variants within the gene locus were utilized as instrumental variables (IVs) to approximate the pharmacological modulation of the drug target protein [23].

The GWAS summary statistics for overall HER2-positive/negative BC and T2D were obtained from the FinnGen research project [24], while the GWAS summary statistics for ER-positive/negative BC were acquired from the Breast Cancer Association Consortium (BCAC) [25]. Due to the nature of the study, no specific ethical approval or written informed consent from participants was deemed necessary.

We subsequently assessed the causal link between genetic variations in metformin targets and the susceptibility to overall BC, and each subtype of BC, using a two-sample MR. Considering that the intended indication for metformin is T2DM, we concurrently investigated the causal relationship between metformin target variations and T2D as a positive control (ref: Guidelines for performing mendelian randomization investigations). Table 1 provides comprehensive details on all the GWAS included in our study.

**Table 1:** Studies used to retrieve summary statistics for the two-sample Mendelian randomization analyses

Trait	Outcome/Exposure	Population	Sample Size	Data Source	Dataset
PRKAB1	Exposure	European	31,684	IEU OpenGWAS	eqtl-a-ENSG00000111725
ETFDH	Exposure	European	26,395	IEU OpenGWAS	eqtl-a-ENSG00000171503
GPD1L	Exposure	European	31,684	IEU OpenGWAS	eqtl-a-ENSG00000152642
T2D	Outcome	European	38,657 cases and 310,131 controls	FinnGen R9	T2D_WIDE
Overall BC	Outcome	European	15,680 cases and 167,189 controls	FinnGen R9	C3_BREAST_EXALLC
ER-positive BC	Outcome	European	4,226 cases and 17,588 controls	IEU OpenGWAS	ieu-a-1134
ER-negative BC	Outcome	European	4,480 cases and 17,588 controls	IEU OpenGWAS	ieu-a-1137
HER2-positive BC	Outcome	European	9,698 cases and 167,017 controls	FinnGen R9	C3_BREAST_ERPLUS_EXALLC
HER2-negative BC	Outcome	European	5,965 cases and 167,017 controls	FinnGen R9	C3_BREAST_ERNEG_EXALLC

### Screening of IVs

We implemented several quality control measures to identify suitable genetic instrumental tools [26]. Initially, we identified single nucleotide polymorphisms (SNPs) that achieved genome-wide significance (*P* < 5.0E − 08). Subsequently, a clumping process was applied, utilizing linkage disequilibrium (LD) estimates from Europeans in the 1000 Genomes project (R2 < 0.1, window size of 500 kb) to ensure the independence of genetic variables [27]. In cases where SNP pairs showed LD R2 values surpassing the threshold (0.1), we retained the SNP with the lower P value.

To detect potential weak instrumental variable bias, we calculated the F-statistic (F = R^2^(n - k − 1)/k (1 - R^2^)), where R^2^, n, and k denote the proportion of variance in exposure explained by selected genetic tools, the sample size of the exposure GWAS, and the number of chosen genetic tools, respectively. An average F-statistic exceeding 10 indicates suitable instrumental variables [28].

Adhering to the two fundamental IV analysis assumptions, we removed SNPs linked to potential confounding variables and adjusted for SNPs exhibiting horizontal pleiotropic effects through consultation with the PhenoScanner database [29].

### MR analysis

In this study, we conducted two separate two-sample MR analyses. Initially, we aligned SNPs to standardize the exposure and outcome data. Subsequently, the inverse-variance weighting (IVW) method was used to assess heterogeneity between SNPs. A p-value greater than 0.05 for the Q-statistic indicated the absence of heterogeneity. Furthermore, for detecting horizontal pleiotropy, we performed the MR-Egger regression intercept test. Finally, based on the evaluation of between-SNP heterogeneity and horizontal pleiotropy, we selected the primary MR method. In order to ensure the robustness of the results, five distinct methods were employed in the present study. The fixed-effect IVW approach was utilized when neither heterogeneity nor pleiotropy were present, while the random-effect IVW method was employed in cases where heterogeneity was observed but pleiotropy was absent [30, 31]. In situations involving pleiotropy, with or without heterogeneity, the MR-Egger regression technique was applied [32, 33]. Additionally, the influence of SNPs was identified using leave-one-out analyses. All statistical analyses were performed using the R program (version 4.2.1), with MR analysis being implemented through the utilization of the TwoSampleMR packages [34]. Both sides of the test were considered statistically significant at 0.05.

## Results

### Genetic instruments for metformin

Three targets of metformin were retrieved from the DrugBank database: PRKAB1, ETFDH and GPD1L, with metformin acting as its inducer, inhibitor and inhibitor, respectively. All F-statistics fell within the range of 49.734 to 7601.343, suggesting that IVs were robust [28]. Further information regarding the SNPs can be found in Supplement Tables S1–16.

### Positive control analysis

**Fig. 2:**
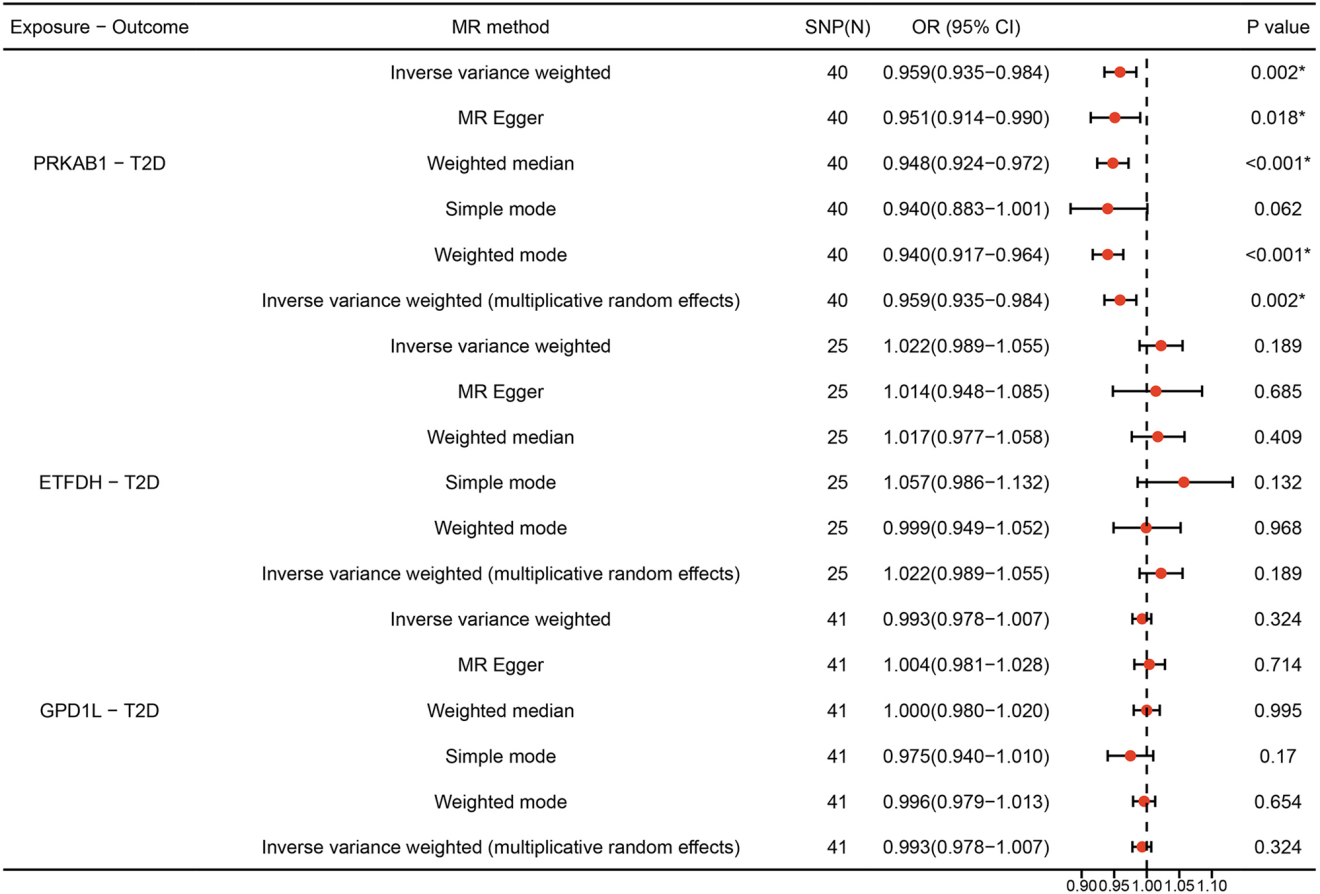
MR estimates derived from the fixed-effect IVW method, MR-Egger regression, weighted median method, weighted-mode method, simple-mode and random-effect IVW method to assess the causal effect between metformin targets and type 2 diabetes (T2D)

Although no substantial causal relationship exists between long-term exposure to functional inhibition of ETFDH or GPD1L and T2D risk (ETFDH: OR[95%] = 1.022 [0.989 to 1.055], *P* = 0.189; GPD1L: OR[95%] = 0.993 [0.978 to 1.007], *P* = 0.324), the anticipated outcome was confirmed by the IVW method, which revealed that overexpression of PRKAB1 with AMP-activated protein kinase activity significantly reduced the risk of T2D (OR[95%] = 0.959 [0.935 to 0.984], *P* = 0.002). Consistency in results was also observed across MR Egger, simple model, weighted model, and MR-PRESSO analyses (Fig. 2).

### The causal relationship between metformin targets and overall BC

**Fig. 3:**
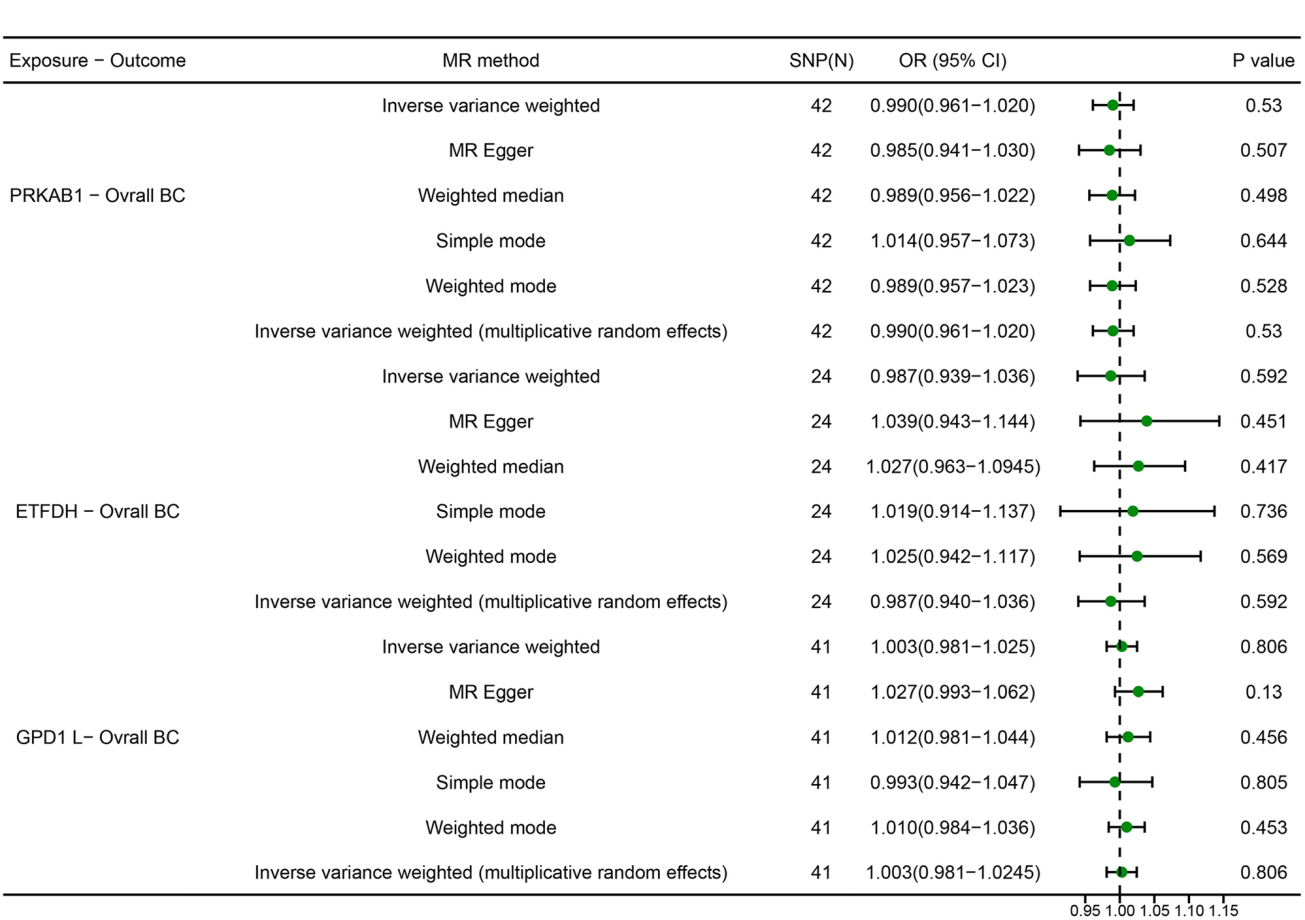
MR estimates derived from the fixed-effect IVW method, MR-Egger regression, weighted median method, weighted-mode method, simple-mode and random-effect IVW method to assess the causal effect between metformin targets and Overall breast cancer (BC).

No significant causal relationship found between inhibition or activation of any metformin target and risk of BC (PRKAB1: OR [95%] = 0.990 [0.961 to 1.020], *P* = 0.530; ETFDH: OR [95%] = 0.987 [0.939 to 1.036], *P* = 0.582; GPD1L: OR [95%] = 1.003 [0.981 to 1.025], *P* = 0.806). Consistency in results was also observed across MR Egger, simple model, weighted model, and MR-PRESSO analyses (Fig. 3)

### The causal relationship between metformin targets and ER-positive/negative BC

**Fig. 4:**
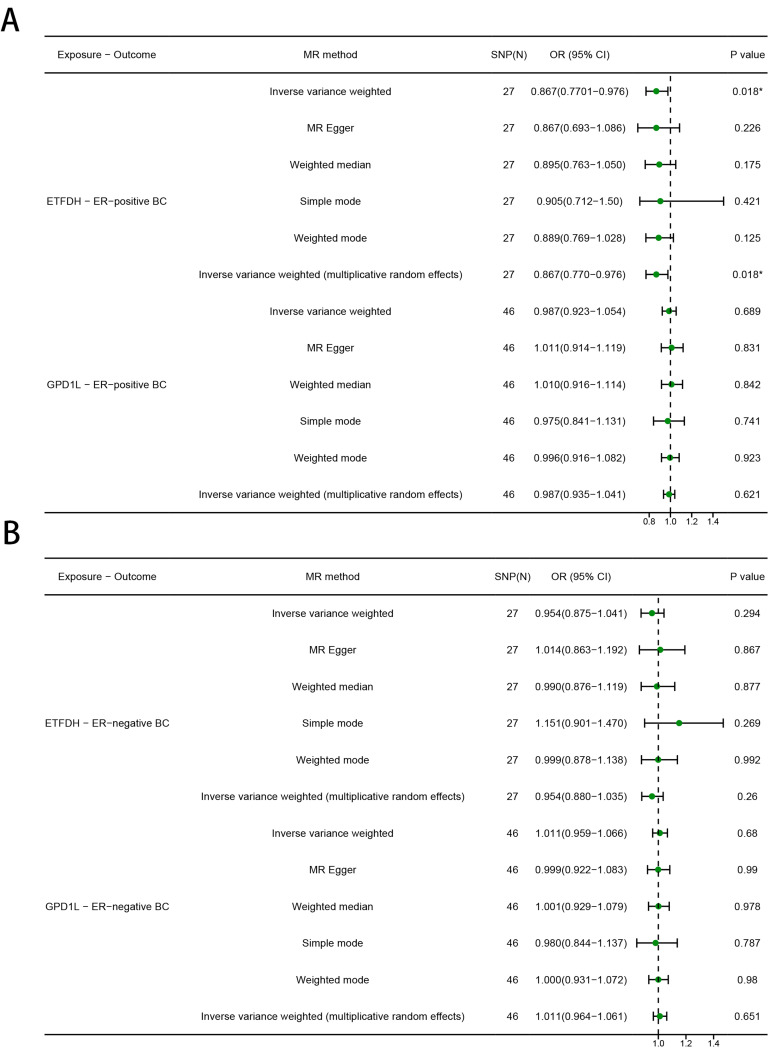
MR estimates derived from the fixed-effect IVW method, MR-Egger regression, weighted median method, weighted-mode method, simple-mode and random-effect IVW method to assess the causal effect between metformin targets and ER (**A**) positive/ (**B**) negative breast cancer (BC)

Reduced risk of ER-positive BC was significantly associated with genetic variants in ETFDH (OR [95%] = 0.867 [0.770 to 0.976], *P* = 0.018), but not with GPD1L (OR [95%] = 0.987 [0.923 to 1.054], *P* = 0.689) and PRKAB1 (No valid genetic instruments were found) (Fig. 4A). For ER-negative BC, no significant causal association was found between any metformin target and BC risk (PRKAB1: No valid genetic instruments were found; ETFDH: OR [95%] = 0.954 [0.875 to 1.041], *P* = 0.294; GPD1L: OR [95%] = 1.011 [0.959 to 1.066], *P* = 0.680) (Fig. 4B). Consistency in results was also observed across MR Egger, simple model, weighted model, and MR-PRESSO analyses. (Fig. 4A, B)

### The causal relationship between metformin targets and HER2-positive/negative BC

**Fig. 5:**
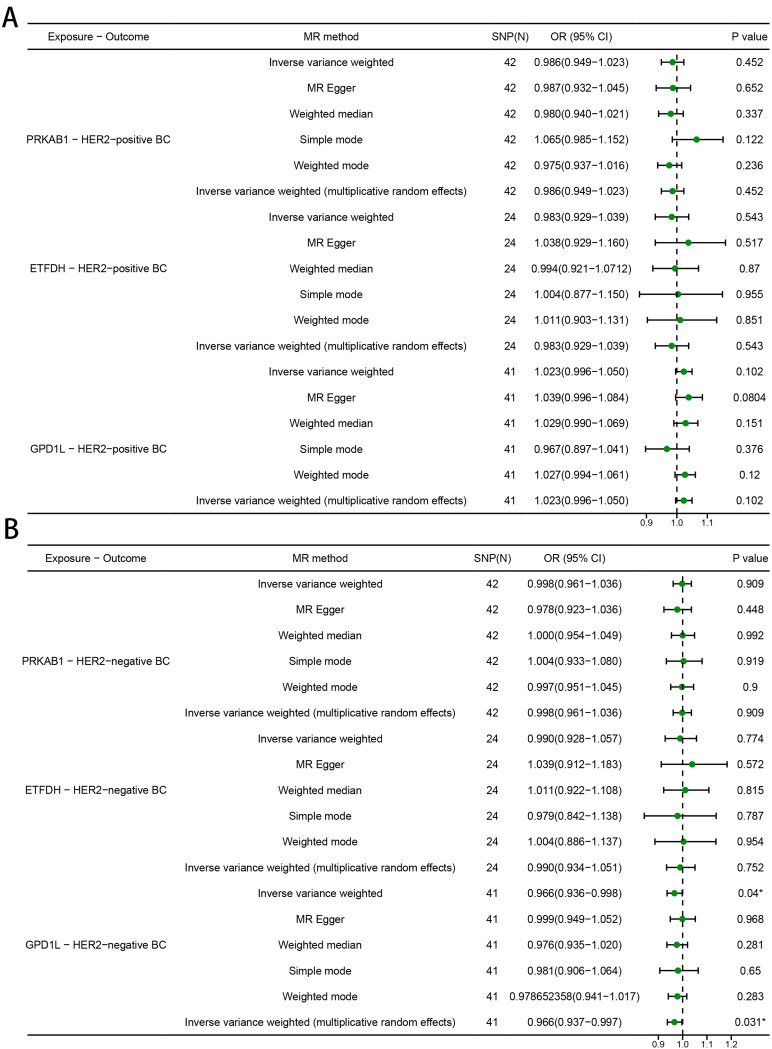
MR estimates derived from the fixed-effect IVW method, MR-Egger regression, weighted median method, weighted-mode method, simple-mode and random-effect IVW method to assess the causal effect between metformin targets and HER2 (**A**) positive/ (**B**) negative breast cancer (BC)

For HER-positive BC, no significant causal association was found between any metformin target and BC risk (PRKAB1: OR [95%] = 0.986 [0.949 to 1.023], *P* = 0.452; ETFDH: OR [95%] = 0.983 [0.929 to 1.039], *P* = 0.543; GPD1L: OR [95%] = 1.023 [0.996 to 1.050], *P* = 0.102) (Fig. 5A). Reduced risk of HER2-negative BC was significantly associated with genetic variants in GPD1L (OR [95%] = 0.966 [0.936 to 0.998], *P* = 0.040), but not with PRKAB1 (OR [95%] = 0.998 [0.961 to 1.036], *P* = 0.909); ETFDH: OR [95%] = 0.990 [0.928 to 1.057], *P* = 0.774 (Fig. 5B). Consistency in results was also observed across MR Egger, simple model, weighted model, and MR-PRESSO analyses (Fig. 5A, B).

### Multiple causal path assessment

To address confounding issues among multiple causal factors, we attempted multivariable Mendelian randomization analysis to simultaneously evaluate the causal pathways between the three targets and BC [35, 36]. However, there was no enough overlap between effective IVs for the three targets and BC, making further analysis infeasible. This suggests that the likelihood of significant causal interactions among the three targets on breast cancer is low.

### Sensitivity analysis

The level of heterogeneity and horizontal pleiotropy was assessed using Cochrane’s Q and MR Egger regression equation, and leave-one-out analysis was conducted to identify influential SNPs (Tables S17, Figure S1-6). Significant heterogeneity was observed when examining the causal relationship between PRKAB1 variation and overall BC, HER2-positive BC, and T2D. Importantly, the random-effect IVW method employed in this study effectively mitigates bias resulting from heterogeneity in the findings [30]. Additionally, no heterogeneity or pleiotropy was detected in other groups analyses.

## Discussion

This is the first MR study to investigate whether genetic variations in metformin targets are associated with the risk of overall BC, as well as ER-positive/negative BC and HER2-positive/negative BC. We retrieved the three key action targets of metformin from the Drugbank database: PRKAB1 [37], ETFDH [38, 39], and GPD1L [40, 41]. Among them, PRKAB1, also known as 5’AMP-activated protein kinase (AMPK) subunit beta-1 or AMPK, which as AMP-activated protein kinase activity, is the most critical target of metformin. AMPK is not solely the primary focus of glucose reduction; moreover, extant research indicates that metformin also depends on its anticancer properties [42]. The prevailing perspective posits that metformin triggers AMPK activation within cancerous cells, thereby instigating metabolic reprogramming and impeding the utilization of nutrient resources, ultimately impeding proliferation [43]. Regrettably, based on robust evidence, leveraging existing large-scale genetic association data on BC risk, our genetic investigation suggests that metformin might exert no protective effects on overall BC, ER-negative BC, and HER2-positive BC within European populations. The findings from this study indicate that there exists no discernible causal link between the functional activation of the star target AMPK (PRKAB1) and the mitigation of breast cancer risk, failing to manifest the anticipated anticancer efficacy. This suggests that despite certain preclinical investigations suggesting metformin’s potential in reducing cancer risk, its translation into the clinical context is not a straightforward process [9, 44].

Furthermore, our research suggests a significant causal association between elevated ETFDH expression as an exposure factor and a reduced probability of developing ER-positive BC. Similarly, a high expression level of GPD1L is correlated with a decreased likelihood of developing HER2-negative BC. These findings suggest that, overall among breast cancer patients, metformin not only fails to mitigate the risk of breast cancer in each subtype, but also possesses the potential to increase the risk of ER-positive BC and HER2-negative BC by inhibiting ETFDH and GPD1L.

This might appear disappointing at a first glance but not unexpected. While initial epidemiologic studies suggested a preventive role of metformin in BC [45, 46], subsequent research and meta-analyses presented conflicting conclusions [47, 48]. Most recently, the largest phase 3 randomized trial investigating metformin as adjuvant therapy for BC, comprising 3,649 women with a 5-year follow-up, revealed no discernible benefits in terms of disease-free survival or overall survival with metformin [10]. Oriana Hoi et al. highlighted in their review [49] that, despite experimental studies supporting metformin’s anticancer effects, many used suprapharmacological doses, reaching plasma levels 10 to 100 times higher than achievable in humans [50]. As such, findings from these studies may not translate to the same effects in humans [42, 50]. Numerous observational clinical studies have demonstrated susceptibility to time-related bias, leading to an overestimation of the drug’s advantages. Thus far, randomized trials investigating metformin as a therapeutic intervention for diverse cancer types have not yielded any evidence of diminished disease-free survival or overall survival rates [49]. Our study is the inaugural investigation into the causal link between metformin target variations and the risk of distinct breast cancer subtypes using drug-target MR stratification. The findings offer fresh evidence contradicting the notion of metformin as a standard adjunctive therapy for BC.

Furthermore, our findings imply that metformin, acting as an ETFDH inhibitor, might potentially exert a promoting effect on ER-positive BC. Previous studies have highlighted that low ETFDH expression correlates significantly with poorer survival in various tumors, including hepatocellular carcinoma and colorectal cancer [51, 52]. Some researchers suggest that although metformin may benefit ER-positive BC patients initially, the effect of long-term use may be reversed [15, 53]. Additionally, several investigations have reported metformin use was associated with an augmented incidence of ER-positive BC, aligning with our study results [54].

Our findings also indicate that metformin may enhance the development of HER 2-negative breast cancer by inhibiting GPD1L. Ye Du et al. have confirmed that the downregulation of GPD1L is linked to metabolic reprogramming in triple-negative breast cancer (TNBC) as a direct downstream target of aerobic glycolysis and oncogenic activity mediated by mir-210-3p [55]. A study indicates that metformin can induce metabolic adaptation in breast cancer cells, increasing resistance to metformin and promoting the accumulation of TNBC-derived BCSCs, which could eventually lead to cancer cell invasion, metastasis, and recurrence [56]. Matthew G. Costales et al. have identified a small molecule called Targapremir-210 in TNBC and observed a significant increase in GPD1L levels in TNBC tumor tissues in the Targapremir-210 treated group, disrupting the hypoxic adaptive response that promotes tumor growth [57]. While specific studies confirming the impact of GPD1L dysregulation on HER2-negative BC are lacking, the clear inhibitory effect of high GPD1L expression on TNBC may suggest evidence for the potential promoting effect of metformin on HER2-negative BC.

Certainly, we need to consider whether metformin interacts with other major BC treatments such as surgery, radiotherapy, chemotherapy, endocrine therapy, and targeted therapy. Currently, no evidence links metformin’s efficacy to surgical intervention. Its radiosensitizing effects have been preliminarily explored in mice, suggesting that while metformin with oxygen microbubbles may enhance short-term radiosensitivity, it does not reverse treatment resistance and may promote metastasis [58]. Although some studies suggest metformin can reverse chemotherapy resistance in vitro, this does not translate effectively in vivo or clinically [59, 60]. Additionally, although some studies have explored combining metformin with endocrine and targeted therapies, evidence shows that metformin promotes the survival of dormant estrogen receptor-positive breast cancer cells by activating AMPK, cautioning against widespread use of AMPK activators [61]. Long-term metformin treatment may also lead to dual resistance to tamoxifen and metformin through the Akt/Snail1/E-cadherin signaling axis [62]. Existing clinical data supporting the inclusion of metformin in BC treatment primarily come from survival analyses of BC patients with diabetic patients [63–65]. This may be because diabetes itself is a high-risk factor for BC [66, 67], and metformin might help combat diabetes-related BC risk while treating diabetes. However, this does not justify expanding its use to non-diabetic populations with normal blood glucose levels.

Not only that, the compliance of metformin cannot be ignored. Metformin is recognized for inducing gastrointestinal side effects, including nausea, diarrhea, and abdominal discomfort, affecting 20–30% of the general population, notably during initial dose adjustment [68, 69]. In a substantial double-blind trial for ER-positive early BC, non-adherence was more prevalent among metformin-treated patients [70]. Consequently, metformin does not appear to be a viable standard complementary treatment for breast cancer due to concerns about both anticancer efficacy and patient compliance.

Our study exhibits notable strengths. Firstly, it pioneers a unique MR investigation comparing metformin and breast cancer (both overall and subtypes) in European populations, filling a gap in the current literature. The two-sample design ensures a causal inference devoid of confounding bias and reverse causation. Moreover, the large F statistic underscores minimal risk of a weak instrument bias. Lastly, we meticulously selected SNPs showing significant associations with the exposure variable but lacking direct associations with the outcome variable. It is worth mentioning that our study, compared to previous epidemiological studies, has a much larger sample size. We not only concluded that there is no significant causal relationship between metformin use and reduced overall breast cancer risk but also suggested that metformin might actually increase the risk of ER-positive and HER2-negative BC by inhibiting ETFDH and GPD1L. Although there is not yet sufficient clinical data to support this finding, much laboratory evidence supports our conclusion.

Despite the aforementioned strengths, it is crucial to acknowledge several inescapable limitations in our study. Firstly, our results should primarily be interpreted as a test of causal association and cannot replace clinical trials in the real-world setting. Secondly, our analyses did not consider the combined efficacy of drug interactions in clinical scenarios. Thirdly, the drug target MR effect estimates mainly correspond to continuous, long-term modulation of drug targets and may not reflect the impact of short-term drug use on breast cancer. Lastly, our MR analysis was limited to the European population due to inadequate GWAS data resources, raising uncertainty about the generalizability of our findings to different ethnic populations. Additionally, due to the limited information in the databases, further stratified analysis may not have been conducted for patients with different clinical-pathological characteristics. Therefore, future research should conduct subgroup analyses on different racial populations, expand the study samples for each breast cancer subtype, and supplement the design with considerations for short-term and long-term medication. A specific and thorough tracking investigation should be implemented to assess short-term and long-term benefits, aiming to derive more comprehensive conclusions.

## Conclusions

To sum up, the main target of metformin, PRKAB1, does not demonstrate a substantial causal association with the risk of BC. Conversely, metformin, acting as an inhibitor of ETFDH and GPD1L, may elevate the risk of developing ER-positive BC and HER2-negative BC. This research presents innovative genetic evidence indicating that metformin may not serve as a promising standard adjunctive therapy for BC patients without T2D.

### Electronic supplementary material

Below is the link to the electronic supplementary material.

Supplementary Material 1

## Data Availability

All data needed to evaluate the conclusions in the paper are present in the paper and/or the Supplementary Materials. Additional data related to this paper may be requested from the authors.

## Abbreviations

BC: Breast cancer
T2D: Type 2 diabetes
MR: Mendelian Randomization
OR: Odds ratio
ER: Estrogen receptor
RCT: Randomized controlled trial
GWAS: Genome-wide association studies
BCAC: Breast Cancer Association Consortium
SNPs: Single nucleotide polymorphisms
LD: Linkage disequilibrium
IVs: Instrumental variables
IVW: Inverse-variance weighting
AMPK: 5’AMP-activated protein kinase
