# Supplementary material for: New evidence: Metformin unsuitable as routine adjuvant for breast cancer: a drug-target mendelian randomization analysis
Source: BMC Cancer. 2024 Jun 6;24:691. doi: 10.1186/s12885-024-12453-w (PMC11155042; doi:10.1186/s12885-024-12453-w)
Supplement: Supplementary file 1 — Supplementary Material 1 [file 12885_2024_12453_MOESM1_ESM.pdf]

Supplementary Materials for

New Evidence: Metformin Unsuitable as Routine Adjuvant for Breast Cancer - A Drug-Target Mendelian Randomization Analysis

Jing-Xuan Xu *et al.*

*Corresponding author:Dr. Yu-Chong Peng Email: pengyuchong2018@126.com

This PDF file includes:
Supplementary Text
Tables S1-S17
Figs. S1 to S6#

Supplementary Table 1. Characteristics of PRKAB1 variants associated with type 2 diabetes (T2D)

Population	SNP	EA	OA	SNP-PRKAB1				SNP-T2D		
				beta	SE	P-value	F	beta	SE	P-value
European	rs1057686	G	A	-0.144856	0.0159405	1.02E-19	174.1965845	0.00204263	0.0109165	0.85157
European	rs11064762	C	T	0.263653	0.0402051	5.46E-11	90.71397488	-0.0352275	0.0290576	0.225384
European	rs11064809	A	G	-0.359108	0.0323506	1.25E-28	259.9295015	-0.0150081	0.0189383	0.428085
European	rs11064834	G	A	0.177554	0.024996	1.22E-12	106.4369648	-0.0378198	0.0234047	0.106116
European	rs11064854	G	A	-0.604448	0.0211658	2.25E-179	1720.362081	-0.0063032	0.0160235	0.694046
European	rs11064994	G	A	0.199057	0.0254252	4.91E-15	129.2990873	-0.0307523	0.0199432	0.123076
European	rs11065015	T	C	-0.340478	0.0376348	1.47E-19	172.6512718	-0.0266699	0.0185297	0.150065
European	rs11065159	G	A	0.278475	0.042538	5.89E-11	90.40433063	0.0471991	0.0448364	0.29248
European	rs112088749	A	G	0.191045	0.0320067	2.39E-09	75.15543343	0.0249352	0.0166458	0.134137
European	rs113004644	T	C	-0.25128	0.0175702	2.14E-46	431.4561238	0.0120523	0.011551	0.296764
European	rs117067019	C	T	0.388195	0.0338807	2.15E-30	276.9267894	0.0297521	0.028581	0.297888
European	rs117076788	T	C	-0.645808	0.0378015	1.95E-65	615.6871767	0.073582	0.0556311	0.185943
European	rs117200164	A	G	-0.577229	0.0375424	2.39E-53	498.6816186	0.0573247	0.035205	0.103458
European	rs117872759	G	A	0.596178	0.0278516	1.19E-101	966.5461546	-0.0607593	0.0177285	0.000609846
European	rs12296843	G	A	-0.175676	0.0140428	6.58E-36	330.1320132	-0.00947866	0.0110557	0.39125
European	rs137937400	A	G	0.195996	0.030045	6.87E-11	89.76796814	0.0469179	0.0245379	0.0558689
European	rs138689173	A	G	-0.319293	0.0454886	2.23E-12	103.9309835	-0.0357634	0.0251037	0.154265
European	rs141007011	C	T	-0.469949	0.0480387	1.34E-22	201.8784935	-0.0108	0.0291159	0.71069
European	rs143544080	A	C	0.918076	0.0453687	4.73E-91	863.8042689	-0.102279	0.025581	6.38E-05
European	rs149857526	T	G	-0.409311	0.0467596	2.07E-18	161.6351493	-0.0573568	0.0320286	0.0733263
European	rs17485664	C	T	1.06226	0.0227316	0	4606.508154	-0.0800291	0.0180781	9.56E-06

Supplementary Table 1. Continued

Population	SNP	EA	OA	SNP-PRKAB1				SNP-T2D		
				beta	SE	P-value	F	beta	SE	P-value
European	rs182726850	G	A	-0.362822	0.0452238	1.03E-15	135.7759209	0.0157332	0.0425629	0.711646
European	rs2464798	G	A	0.285394	0.0133174	6.99E-102	968.7717428	-0.0248205	0.00980165	0.0113324
European	rs2524393	G	A	-0.152407	0.0224839	1.21E-11	96.92521409	-0.0269857	0.0141512	0.0565275
European	rs34179846	C	T	0.235414	0.0203425	5.68E-31	282.5043508	-0.0207343	0.0137898	0.132684
European	rs35811623	G	A	0.192009	0.0340979	1.79E-08	66.8897268	-0.0200829	0.0199802	0.314828
European	rs3902094	A	G	0.565209	0.0448337	1.94E-36	335.2576808	-0.0614724	0.0265512	0.0205997
European	rs3932409	T	C	0.155719	0.0139577	6.66E-29	262.5604323	-0.00808932	0.00949233	0.394106
European	rs4465416	T	G	0.0911742	0.0119307	2.14E-14	123.1927564	-0.000872247	0.00827735	0.916076
European	rs528708	G	T	0.143759	0.0158041	9.35E-20	174.5420969	0.00657508	0.0113804	0.56343
European	rs73217238	C	T	0.104388	0.0147619	1.53E-12	105.48385	0.0322675	0.0106859	0.00253075
European	rs73221259	A	G	1.04417	0.0449267	1.73E-119	1139.470312	-0.0290776	0.0214669	0.175567
European	rs73221277	A	G	-0.660658	0.033506	1.52E-86	820.1243233	0.00535933	0.0232856	0.81797
European	rs76382809	T	C	-0.40127	0.0383205	1.17E-25	231.3028122	-0.0409889	0.037379	0.272826
European	rs76490401	G	A	0.188095	0.0339044	2.89E-08	64.92530533	0.00667407	0.0240413	0.781313
European	rs77051199	T	C	0.292949	0.037859	1.01E-14	126.3039409	0.0446365	0.0327573	0.172995
European	rs7956304	A	G	0.129969	0.0143759	1.56E-19	172.4180386	-0.0232238	0.00977381	0.0174952
European	rs804441	T	C	0.16195	0.0130092	1.42E-35	326.911158	0.0118295	0.00840438	0.159267
European	rs804460	T	G	-0.0923487	0.0119227	9.51E-15	126.5555895	0.0373968	0.00826893	6.11E-06
European	rs884033	G	A	0.139006	0.0120504	8.75E-31	280.6963254	-0.0270413	0.00844581	0.00136603

SNP, single nucleotide polymorphisms; EA, effect allele; OA, other allele; SE, standard error; F, F-statistics.

Supplementary Table 2. Characteristics of PRKAB1 variants associated with Overall breast cancer (BC)

Population	SNP	EA	OA	SNP-PRKAB1				SNP-Overall BC		
				beta	SE	P-value	F	beta	SE	P-value
European	rs1057686	G	A	-0.144856	0.0159405	1.02E-19	174.1965845	0.0015833	0.0165319	0.923702
European	rs11064762	C	T	0.263653	0.0402051	5.46E-11	90.71397488	0.00697519	0.0448263	0.876345
European	rs11064809	A	G	-0.359108	0.0323506	1.25E-28	259.9295015	-0.0137413	0.0289264	0.634756
European	rs11064834	G	A	0.177554	0.024996	1.22E-12	106.4369648	0.0269744	0.0354886	0.447202
European	rs11064854	G	A	-0.604448	0.0211658	2.25E-179	1720.362081	0.0522369	0.0243962	0.0322582
European	rs11064994	G	A	0.199057	0.0254252	4.91E-15	129.2990873	0.0399033	0.0302624	0.18731
European	rs11065015	T	C	-0.340478	0.0376348	1.47E-19	172.6512718	0.0218253	0.0283094	0.440732
European	rs11065159	G	A	0.278475	0.042538	5.89E-11	90.40433063	0.0444906	0.0674207	0.509321
European	rs112088749	A	G	0.191045	0.0320067	2.39E-09	75.15543343	-0.0506543	0.0251066	0.0436365
European	rs113004644	T	C	-0.25128	0.0175702	2.14E-46	431.4561238	-0.00725914	0.0175228	0.678677
European	rs117067019	C	T	0.388195	0.0338807	2.15E-30	276.9267894	0.0450188	0.0437731	0.303735
European	rs117076788	T	C	-0.645808	0.0378015	1.95E-65	615.6871767	-0.0474689	0.0847477	0.575398
European	rs117200164	A	G	-0.577229	0.0375424	2.39E-53	498.6816186	0.0163782	0.0535226	0.759602
European	rs117872759	G	A	0.596178	0.0278516	1.19E-101	966.5461546	-0.00285042	0.0268529	0.915464
European	rs12296843	G	A	-0.175676	0.0140428	6.58E-36	330.1320132	-0.00179552	0.016789	0.914831
European	rs137937400	A	G	0.195996	0.030045	6.87E-11	89.76796814	0.0705589	0.0368381	0.0554447
European	rs138689173	A	G	-0.319293	0.0454886	2.23E-12	103.9309835	-0.0317634	0.0379306	0.402364
European	rs141007011	C	T	-0.469949	0.0480387	1.34E-22	201.8784935	-0.0444643	0.0447854	0.320792
European	rs143544080	A	C	0.918076	0.0453687	4.73E-91	863.8042689	-0.0156269	0.038345	0.683617
European	rs149857526	T	G	-0.409311	0.0467596	2.07E-18	161.6351493	-0.0204196	0.0486744	0.67484
European	rs17485664	C	T	1.06226	0.0227316	0	4606.508154	-0.0118767	0.0270832	0.661005

Supplementary Table 2. Continued

Population	SNP	EA	OA	SNP-PRKAB1				SNP-Overall BC		
				beta	SE	P-value	F	beta	SE	P-value
European	rs182726850	G	A	-0.362822	0.0452238	1.03E-15	135.7759209	0.0232882	0.063871	0.7154
European	rs202983	A	G	-0.117269	0.0134383	2.63E-18	160.6386515	-0.00562055	0.0147895	0.703918
European	rs2464798	G	A	0.285394	0.0133174	6.99E-102	968.7717428	-0.0149507	0.0149378	0.316894
European	rs2524393	G	A	-0.152407	0.0224839	1.21E-11	96.92521409	-0.0156481	0.0215039	0.466803
European	rs34179846	C	T	0.235414	0.0203425	5.68E-31	282.5043508	0.0257941	0.0211462	0.222541
European	rs35811623	G	A	0.192009	0.0340979	1.79E-08	66.8897268	-0.0505968	0.0303794	0.0958142
European	rs3902094	A	G	0.565209	0.0448337	1.94E-36	335.2576808	0.00987984	0.0404259	0.806926
European	rs3932409	T	C	0.155719	0.0139577	6.66E-29	262.5604323	-0.0417608	0.0144094	0.00375362
European	rs4465416	T	G	0.0911742	0.0119307	2.14E-14	123.1927564	-0.00906703	0.0125817	0.471125
European	rs4767921	G	A	0.10331	0.0124791	1.25E-16	144.5725179	0.013879	0.0135326	0.305082
European	rs528708	G	T	0.143759	0.0158041	9.35E-20	174.5420969	0.0252258	0.0172303	0.143183
European	rs73217238	C	T	0.104388	0.0147619	1.53E-12	105.48385	-0.00794858	0.016231	0.624335
European	rs73221259	A	G	1.04417	0.0449267	1.73E-119	1139.470312	-0.0129177	0.0325867	0.691802
European	rs73221277	A	G	-0.660658	0.033506	1.52E-86	820.1243233	-0.0482855	0.0348149	0.165466
European	rs76382809	T	C	-0.40127	0.0383205	1.17E-25	231.3028122	0.16496	0.054962	0.00268788
European	rs76490401	G	A	0.188095	0.0339044	2.89E-08	64.92530533	-0.0443095	0.0364227	0.223781
European	rs77051199	T	C	0.292949	0.037859	1.01E-14	126.3039409	-0.155403	0.0514437	0.00252069
European	rs7956304	A	G	0.129969	0.0143759	1.56E-19	172.4180386	0.0518965	0.0147614	0.000438622
European	rs804441	T	C	0.16195	0.0130092	1.42E-35	326.911158	0.0053785	0.0127551	0.673263
European	rs804460	T	G	-0.0923487	0.0119227	9.51E-15	126.5555895	0.015202	0.0126103	0.228001
European	rs884033	G	A	0.139006	0.0120504	8.75E-31	280.6963254	-0.00704927	0.012818	0.582353

SNP, single nucleotide polymorphisms; EA, effect allele; OA, other allele; SE, standard error; F, F-statistics.

Supplementary Table 3. Characteristics of PRKAB1 variants associated with HER2-positive breast cancer (HER2+ BC)

Population	SNP	EA	OA	SNP-PRKAB1				SNP-HER2+ BC		
				beta	SE	P-value	F	beta	SE	P-value
European	rs1057686	G	A	-0.144856	0.0159405	1.02E-19	174.1965845	-0.00628433	0.0203984	0.758022
European	rs11064762	C	T	0.263653	0.0402051	5.46E-11	90.71397488	-0.00253057	0.0553672	0.963545
European	rs11064809	A	G	-0.359108	0.0323506	1.25E-28	259.9295015	-0.0273545	0.0357501	0.444177
European	rs11064834	G	A	0.177554	0.024996	1.22E-12	106.4369648	0.0119176	0.0439606	0.786318
European	rs11064854	G	A	-0.604448	0.0211658	2.25E-179	1720.362081	0.0488784	0.0301403	0.104869
European	rs11064994	G	A	0.199057	0.0254252	4.91E-15	129.2990873	0.033176	0.0374246	0.375361
European	rs11065015	T	C	-0.340478	0.0376348	1.47E-19	172.6512718	0.0069501	0.0350801	0.842951
European	rs11065159	G	A	0.278475	0.042538	5.89E-11	90.40433063	0.0717589	0.0832042	0.388444
European	rs112088749	A	G	0.191045	0.0320067	2.39E-09	75.15543343	-0.0653213	0.0309885	0.0350372
European	rs113004644	T	C	-0.25128	0.0175702	2.14E-46	431.4561238	-0.00197096	0.0216437	0.927442
European	rs117067019	C	T	0.388195	0.0338807	2.15E-30	276.9267894	0.0721087	0.0540878	0.182473
European	rs117076788	T	C	-0.645808	0.0378015	1.95E-65	615.6871767	-0.0350178	0.105051	0.738877
European	rs117200164	A	G	-0.577229	0.0375424	2.39E-53	498.6816186	0.0488164	0.0659842	0.45941
European	rs117872759	G	A	0.596178	0.0278516	1.19E-101	966.5461546	-0.0174289	0.0330594	0.598055
European	rs12296843	G	A	-0.175676	0.0140428	6.58E-36	330.1320132	-0.00336807	0.0207429	0.871013
European	rs137937400	A	G	0.195996	0.030045	6.87E-11	89.76796814	0.0735276	0.0456029	0.106887
European	rs138689173	A	G	-0.319293	0.0454886	2.23E-12	103.9309835	-0.0508371	0.0467648	0.277001
European	rs141007011	C	T	-0.469949	0.0480387	1.34E-22	201.8784935	-0.0672277	0.0551753	0.223057
European	rs143544080	A	C	0.918076	0.0453687	4.73E-91	863.8042689	-0.0321295	0.0471926	0.495988
European	rs149857526	T	G	-0.409311	0.0467596	2.07E-18	161.6351493	0.0333471	0.0599222	0.577864
European	rs17485664	C	T	1.06226	0.0227316	0	4606.508154	-0.019134	0.0333997	0.566727

Supplementary Table 3. Continued

Population	SNP	EA	OA	SNP-PRKAB1				SNP-HER2+ BC		
				beta	SE	P-value	F	beta	SE	P-value
European	rs182726850	G	A	-0.362822	0.0452238	1.03E-15	135.7759209	-0.0248881	0.0787993	0.752123
European	rs202983	A	G	-0.117269	0.0134383	2.63E-18	160.6386515	-0.0153893	0.0182715	0.399643
European	rs2464798	G	A	0.285394	0.0133174	6.99E-102	968.7717428	-0.0327878	0.0184462	0.0754884
European	rs2524393	G	A	-0.152407	0.0224839	1.21E-11	96.92521409	-0.0177475	0.0265857	0.504416
European	rs34179846	C	T	0.235414	0.0203425	5.68E-31	282.5043508	0.0161207	0.0261451	0.537508
European	rs35811623	G	A	0.192009	0.0340979	1.79E-08	66.8897268	-0.135635	0.0387956	0.000472009
European	rs3902094	A	G	0.565209	0.0448337	1.94E-36	335.2576808	0.0479516	0.0498981	0.336557
European	rs3932409	T	C	0.155719	0.0139577	6.66E-29	262.5604323	-0.0582251	0.0178258	0.00108948
European	rs4465416	T	G	0.0911742	0.0119307	2.14E-14	123.1927564	-0.00865485	0.0155532	0.577891
European	rs4767921	G	A	0.10331	0.0124791	1.25E-16	144.5725179	0.0104386	0.0167186	0.532385
European	rs528708	G	T	0.143759	0.0158041	9.35E-20	174.5420969	0.027927	0.0212861	0.189527
European	rs73217238	C	T	0.104388	0.0147619	1.53E-12	105.48385	0.00508067	0.0200735	0.800188
European	rs73221259	A	G	1.04417	0.0449267	1.73E-119	1139.470312	-0.024364	0.0402854	0.545322
European	rs73221277	A	G	-0.660658	0.033506	1.52E-86	820.1243233	-0.0959129	0.0430083	0.0257407
European	rs76382809	T	C	-0.40127	0.0383205	1.17E-25	231.3028122	0.0994738	0.0711626	0.162162
European	rs76490401	G	A	0.188095	0.0339044	2.89E-08	64.92530533	0.0309938	0.0449869	0.490854
European	rs77051199	T	C	0.292949	0.037859	1.01E-14	126.3039409	-0.202911	0.0645028	0.00165649
European	rs7956304	A	G	0.129969	0.0143759	1.56E-19	172.4180386	0.0423976	0.018375	0.0210354
European	rs804441	T	C	0.16195	0.0130092	1.42E-35	326.911158	0.0122025	0.0157512	0.438516
European	rs804460	T	G	-0.0923487	0.0119227	9.51E-15	126.5555895	0.0293735	0.0155842	0.0594525
European	rs884033	G	A	0.139006	0.0120504	8.75E-31	280.6963254	-0.015447	0.0158282	0.329105

SNP, single nucleotide polymorphisms; EA, effect allele; OA, other allele; SE, standard error; F, F-statistics.

Supplementary Table 4. Characteristics of PRKAB1 variants associated with HER2-negative breast cancer (HER2- BC)

Population	SNP	EA	OA	SNP-PRKAB1				SNP-HER- BC		
				beta	SE	P-value	F	beta	SE	P-value
European	rs1057686	G	A	-0.144856	0.0159405	1.02E-19	174.1965845	0.0126025	0.0256106	0.622661
European	rs11064762	C	T	0.263653	0.0402051	5.46E-11	90.71397488	0.0278187	0.0694877	0.688906
European	rs11064809	A	G	-0.359108	0.0323506	1.25E-28	259.9295015	0.00281035	0.0447275	0.9499
European	rs11064834	G	A	0.177554	0.024996	1.22E-12	106.4369648	0.0607174	0.0547899	0.267781
European	rs11064854	G	A	-0.604448	0.0211658	2.25E-179	1720.362081	0.054537	0.0378412	0.149526
European	rs11064994	G	A	0.199057	0.0254252	4.91E-15	129.2990873	0.0508602	0.0466977	0.276094
European	rs11065015	T	C	-0.340478	0.0376348	1.47E-19	172.6512718	0.0440622	0.0437345	0.313697
European	rs11065159	G	A	0.278475	0.042538	5.89E-11	90.40433063	-0.0143508	0.104249	0.890511
European	rs112088749	A	G	0.191045	0.0320067	2.39E-09	75.15543343	-0.0241436	0.0388675	0.534482
European	rs113004644	T	C	-0.25128	0.0175702	2.14E-46	431.4561238	-0.0195908	0.0270837	0.46947
European	rs117067019	C	T	0.388195	0.0338807	2.15E-30	276.9267894	-0.010332	0.067767	0.878821
European	rs117076788	T	C	-0.645808	0.0378015	1.95E-65	615.6871767	-0.0622477	0.131388	0.635663
European	rs117200164	A	G	-0.577229	0.0375424	2.39E-53	498.6816186	-0.0229582	0.0830797	0.782287
European	rs117872759	G	A	0.596178	0.0278516	1.19E-101	966.5461546	0.0111415	0.041743	0.789542
European	rs12296843	G	A	-0.175676	0.0140428	6.58E-36	330.1320132	-0.00347531	0.025944	0.893439
European	rs137937400	A	G	0.195996	0.030045	6.87E-11	89.76796814	0.0680291	0.0570271	0.232899
European	rs138689173	A	G	-0.319293	0.0454886	2.23E-12	103.9309835	-0.00139011	0.0586813	0.981101
European	rs141007011	C	T	-0.469949	0.0480387	1.34E-22	201.8784935	-0.0269116	0.0692043	0.697371
European	rs143544080	A	C	0.918076	0.0453687	4.73E-91	863.8042689	0.00396737	0.0594288	0.946774
European	rs149857526	T	G	-0.409311	0.0467596	2.07E-18	161.6351493	-0.111969	0.0755614	0.138387
European	rs17485664	C	T	1.06226	0.0227316	0	4606.508154	-0.005765	0.0418954	0.890553

Supplementary Table 4. Continued

Population	SNP	EA	OA	SNP-PRKAB1				SNP-HER2+ BC		
				beta	SE	P-value	F	beta	SE	P-value
European	rs182726850	G	A	-0.362822	0.0452238	1.03E-15	135.7759209	0.0865798	0.0987518	0.380626
European	rs202983	A	G	-0.117269	0.0134383	2.63E-18	160.6386515	0.00692007	0.0228979	0.762488
European	rs2464798	G	A	0.285394	0.0133174	6.99E-102	968.7717428	0.0182135	0.0231361	0.431147
European	rs2524393	G	A	-0.152407	0.0224839	1.21E-11	96.92521409	-0.0141369	0.0333139	0.671307
European	rs34179846	C	T	0.235414	0.0203425	5.68E-31	282.5043508	0.0415035	0.0327937	0.20566
European	rs35811623	G	A	0.192009	0.0340979	1.79E-08	66.8897268	0.0773917	0.0468735	0.0987234
European	rs3902094	A	G	0.565209	0.0448337	1.94E-36	335.2576808	-0.055386	0.0625987	0.376276
European	rs3932409	T	C	0.155719	0.0139577	6.66E-29	262.5604323	-0.0171416	0.0221909	0.439841
European	rs4465416	T	G	0.0911742	0.0119307	2.14E-14	123.1927564	-0.0118544	0.019463	0.542477
European	rs4767921	G	A	0.10331	0.0124791	1.25E-16	144.5725179	0.0185472	0.0209213	0.375337
European	rs528708	G	T	0.143759	0.0158041	9.35E-20	174.5420969	0.0235107	0.0266518	0.377701
European	rs73217238	C	T	0.104388	0.0147619	1.53E-12	105.48385	-0.022269	0.0250774	0.374536
European	rs73221259	A	G	1.04417	0.0449267	1.73E-119	1139.470312	0.00364518	0.0504105	0.942355
European	rs73221277	A	G	-0.660658	0.033506	1.52E-86	820.1243233	0.0285181	0.0535142	0.594098
European	rs76382809	T	C	-0.40127	0.0383205	1.17E-25	231.3028122	0.270511	0.0822331	0.00100341
European	rs76490401	G	A	0.188095	0.0339044	2.89E-08	64.92530533	-0.169612	0.0585633	0.00377694
European	rs77051199	T	C	0.292949	0.037859	1.01E-14	126.3039409	-0.07966	0.0761934	0.295793
European	rs7956304	A	G	0.129969	0.0143759	1.56E-19	172.4180386	0.064325	0.0227305	0.00465629
European	rs804441	T	C	0.16195	0.0130092	1.42E-35	326.911158	-0.00619398	0.0197812	0.754185
European	rs804460	T	G	-0.0923487	0.0119227	9.51E-15	126.5555895	-0.00391211	0.0195379	0.841299
European	rs884033	G	A	0.139006	0.0120504	8.75E-31	280.6963254	0.00398161	0.0198073	0.840685

SNP, single nucleotide polymorphisms; EA, effect allele; OA, other allele; SE, standard error; F, F-statistics.

Supplementary Table 5. Characteristics of ETFDH variants associated with type 2 diabetes (T2D)

Population	SNP	EA	OA	SNP-ETFDH				SNP-T2D		
				beta	SE	P-value	F	beta	SE	P-value
European	rs10517688	T	C	-0.0819387	0.0135608	1.52E-09	52.90825329	-0.000705908	0.00893125	0.937002
European	rs112507704	A	G	0.259624	0.0203679	3.25E-37	235.4586899	0.00524885	0.0134852	0.697106
European	rs114481283	A	C	0.353578	0.0475341	1.02E-13	80.18206953	-0.0738224	0.053872	0.170584
European	rs115035697	T	C	0.239274	0.0392878	1.13E-09	53.75205699	0.0788761	0.0271817	0.00371014
European	rs11559290	T	C	0.329702	0.0178501	3.56E-76	494.4038596	0.0124878	0.0150148	0.40558
European	rs115877139	G	A	0.296371	0.0372935	1.91E-15	91.5217712	0.0309526	0.0292207	0.289476
European	rs116363527	T	C	-0.399579	0.0308305	2.05E-38	243.4231968	0.0218867	0.0233635	0.348868
European	rs116415754	T	C	-0.286518	0.0317616	1.87E-19	117.9284524	-0.0605989	0.0318345	0.0569679
European	rs12649106	T	C	-0.0936049	0.0124557	5.69E-14	81.84216381	-0.00968099	0.00843625	0.251156
European	rs144423926	A	C	0.444212	0.0488134	9.02E-20	120.0108932	-0.0103076	0.0276121	0.708925
European	rs148935601	T	G	0.408853	0.042469	6.14E-22	134.310262	0.0603452	0.031892	0.0584682
European	rs151287934	T	C	0.293484	0.0462741	2.26E-10	58.29223855	0.0263819	0.0412962	0.522925
European	rs191462603	T	C	0.42587	0.0467444	8.19E-20	120.2858633	0.0378054	0.025966	0.145404
European	rs36028675	C	T	0.182312	0.0144351	1.45E-36	231.1565446	-0.00214212	0.0095554	0.822618
European	rs505986	A	G	-0.0772975	0.0122345	2.65E-10	57.84664551	-0.000968641	0.00837576	0.907931
European	rs55960593	T	C	-0.182379	0.012231	2.79E-50	322.2119517	0.00794043	0.0085925	0.355428
European	rs62351210	G	A	0.346526	0.0376388	3.37E-20	122.83379	0.0609097	0.0329456	0.0644867
European	rs62352866	A	G	0.153868	0.0121683	1.19E-36	231.7146512	0.0108406	0.00818086	0.185131
European	rs669271	A	C	-0.159725	0.012321	1.97E-38	243.5413298	-0.00264291	0.00843293	0.753973
European	rs72691475	A	G	0.282003	0.0481381	4.68E-09	49.73353549	0.0308996	0.0261384	0.237144
European	rs72971304	T	C	0.34466	0.038047	1.32E-19	118.9214506	0.0328151	0.0382536	0.390986

Supplementary Table 5. Continued

Population	SNP	EA	OA	SNP-ETFDH			F	SNP-T2D		
				beta	SE	P-value		beta	SE	P-value
European	rs76465512	A	G	-0.319234	0.0408975	5.92E-15	88.29655078	-0.0235807	0.0461597	0.609455
European	rs77019759	A	G	-0.167177	0.0245614	1.00E-11	67.13737647	0.0223637	0.0155943	0.151546
European	rs77499728	C	T	-0.285343	0.0375961	3.21E-14	83.47713505	-0.00486534	0.0191968	0.799924
European	rs78628731	A	G	0.500249	0.0421429	1.69E-32	204.1941765	-0.0284165	0.019259	0.14008

SNP, single nucleotide polymorphisms; EA, effect allele; OA, other allele; SE, standard error; F, F-statistics.

Supplementary Table 6. Characteristics of ETFDH variants associated with Overall breast cancer (BC)

Population	SNP	EA	OA	SNP-ETFDH				SNP-Overall BC		
				beta	SE	P-value	F	beta	SE	P-value
European	rs10517688	T	C	-0.0819387	0.0135608	1.52E-09	52.90825329	-0.0196125	0.0135879	0.148915
European	rs112507704	A	G	0.259624	0.0203679	3.25E-37	235.4586899	0.0135055	0.0205691	0.511443
European	rs114481283	A	C	0.353578	0.0475341	1.02E-13	80.18206953	-0.086224	0.0810448	0.287372
European	rs115035697	T	C	0.239274	0.0392878	1.13E-09	53.75205699	-0.0255869	0.0417993	0.540447
European	rs11559290	T	C	0.329702	0.0178501	3.56E-76	494.4038596	-0.0171161	0.0228212	0.453249
European	rs115877139	G	A	0.296371	0.0372935	1.91E-15	91.5217712	-0.050976	0.0439642	0.246257
European	rs116363527	T	C	-0.399579	0.0308305	2.05E-38	243.4231968	-0.0151239	0.0353943	0.669162
European	rs116415754	T	C	-0.286518	0.0317616	1.87E-19	117.9284524	0.00160905	0.0484884	0.973528
European	rs12649106	T	C	-0.0936049	0.0124557	5.69E-14	81.84216381	0.00837074	0.0128262	0.513995
European	rs144423926	A	C	0.444212	0.0488134	9.02E-20	120.0108932	-0.00861305	0.0424123	0.839073
European	rs148935601	T	G	0.408853	0.042469	6.14E-22	134.310262	0.104845	0.0489114	0.0320679
European	rs151287934	T	C	0.293484	0.0462741	2.26E-10	58.29223855	0.115426	0.061888	0.0621699
European	rs191462603	T	C	0.42587	0.0467444	8.19E-20	120.2858633	0.0149209	0.0396085	0.706391
European	rs36028675	C	T	0.182312	0.0144351	1.45E-36	231.1565446	0.0103651	0.0145126	0.475093
European	rs505986	A	G	-0.0772975	0.0122345	2.65E-10	57.84664551	0.0106942	0.0127126	0.400221
European	rs55960593	T	C	-0.182379	0.012231	2.79E-50	322.2119517	0.0280745	0.0130769	0.0318024
European	rs62351210	G	A	0.346526	0.0376388	3.37E-20	122.83379	-0.0254063	0.0498826	0.610527
European	rs62352866	A	G	0.153868	0.0121683	1.19E-36	231.7146512	-0.0351735	0.0124271	0.00464922
European	rs72691475	A	G	0.282003	0.0481381	4.68E-09	49.73353549	0.0275902	0.0397466	0.487587
European	rs72971304	T	C	0.34466	0.038047	1.32E-19	118.9214506	-0.0641483	0.0581736	0.270155
European	rs76465512	A	G	-0.319234	0.0408975	5.92E-15	88.29655078	-0.0478235	0.0679418	0.481502

Supplementary Table 6. Continued

Population	SNP	EA	OA	SNP-ETFDH				SNP-Overall BC		
				beta	SE	P-value	F	beta	SE	P-value
European	rs77019759	A	G	-0.167177	0.0245614	1.00E-11	67.13737647	-0.0116015	0.023554	0.62233
European	rs77499728	C	T	-0.285343	0.0375961	3.21E-14	83.47713505	-0.00299456	0.0293134	0.918632
European	rs78628731	A	G	0.500249	0.0421429	1.69E-32	204.1941765	0.0180857	0.0292508	0.536379

SNP, single nucleotide polymorphisms; EA, effect allele; OA, other allele; SE, standard error; F, F-statistics.

Supplementary Table 7. Characteristics of ETFDH variants associated with HER2-positive breast cancer (HER2+ BC)

Population	SNP	EA	OA	SNP-ETFDH				SNP-HER2+ BC		
				beta	SE	P-value	F	beta	SE	P-value
European	rs10517688	T	C	-0.0819387	0.0135608	1.52E-09	52.90825329	-0.00871295	0.0167928	0.603865
European	rs112507704	A	G	0.259624	0.0203679	3.25E-37	235.4586899	-0.00694339	0.0254462	0.784957
European	rs114481283	A	C	0.353578	0.0475341	1.02E-13	80.18206953	-0.0429962	0.100196	0.667835
European	rs115035697	T	C	0.239274	0.0392878	1.13E-09	53.75205699	-0.0293651	0.0516677	0.569801
European	rs11559290	T	C	0.329702	0.0178501	3.56E-76	494.4038596	-0.0304406	0.0282099	0.280554
European	rs115877139	G	A	0.296371	0.0372935	1.91E-15	91.5217712	-0.0753858	0.0542911	0.16497
European	rs116363527	T	C	-0.399579	0.0308305	2.05E-38	243.4231968	-0.026281	0.0436869	0.547456
European	rs116415754	T	C	-0.286518	0.0317616	1.87E-19	117.9284524	-0.0284722	0.0600032	0.635135
European	rs12649106	T	C	-0.0936049	0.0124557	5.69E-14	81.84216381	0.013604	0.0158406	0.390446
European	rs144423926	A	C	0.444212	0.0488134	9.02E-20	120.0108932	0.0066838	0.0524972	0.898689
European	rs148935601	T	G	0.408853	0.042469	6.14E-22	134.310262	0.101307	0.0606912	0.0950736
European	rs151287934	T	C	0.293484	0.0462741	2.26E-10	58.29223855	0.137187	0.076568	0.0731813
European	rs191462603	T	C	0.42587	0.0467444	8.19E-20	120.2858633	0.00298244	0.0488029	0.95127
European	rs36028675	C	T	0.182312	0.0144351	1.45E-36	231.1565446	0.0187776	0.0179322	0.295035
European	rs505986	A	G	-0.0772975	0.0122345	2.65E-10	57.84664551	0.000988367	0.0157007	0.949806
European	rs55960593	T	C	-0.182379	0.012231	2.79E-50	322.2119517	0.0282301	0.0161558	0.0805731
European	rs62351210	G	A	0.346526	0.0376388	3.37E-20	122.83379	-0.0094339	0.0615206	0.878126
European	rs62352866	A	G	0.153868	0.0121683	1.19E-36	231.7146512	-0.0390264	0.0153393	0.0109527
European	rs72691475	A	G	0.282003	0.0481381	4.68E-09	49.73353549	0.00664815	0.0489896	0.892054
European	rs72971304	T	C	0.34466	0.038047	1.32E-19	118.9214506	-0.0734137	0.0716968	0.305861
European	rs76465512	A	G	-0.319234	0.0408975	5.92E-15	88.29655078	-0.143706	0.0847153	0.0898214

Supplementary Table 7. Continued

Population	SNP	EA	OA	SNP-ETFDH				SNP-HER2+ BC		
				beta	SE	P-value	F	beta	SE	P-value
European	rs77019759	A	G	-0.167177	0.0245614	1.00E-11	67.13737647	-0.0279381	0.0290692	0.336508
European	rs77499728	C	T	-0.285343	0.0375961	3.21E-14	83.47713505	0.008913	0.0362276	0.805661
European	rs78628731	A	G	0.500249	0.0421429	1.69E-32	204.1941765	0.0191592	0.0360862	0.595469

SNP, single nucleotide polymorphisms; EA, effect allele; OA, other allele; SE, standard error; F, F-statistics.

Supplementary Table 8. Characteristics of ETFDH variants associated with HER2-negative breast cancer (HER2- BC)

Population	SNP	EA	OA	SNP-ETFDH				SNP-HER2- BC		
				beta	SE	P-value	F	beta	SE	P-value
European	rs10517688	T	C	-0.0819387	0.0135608	1.52E-09	52.90825329	-0.0350229	0.0210084	0.0954971
European	rs112507704	A	G	0.259624	0.0203679	3.25E-37	235.4586899	0.0445766	0.0317777	0.160687
European	rs114481283	A	C	0.353578	0.0475341	1.02E-13	80.18206953	-0.138889	0.124724	0.265462
European	rs115035697	T	C	0.239274	0.0392878	1.13E-09	53.75205699	0.00240199	0.0649811	0.970513
European	rs11559290	T	C	0.329702	0.0178501	3.56E-76	494.4038596	0.00449559	0.0352705	0.898576
European	rs115877139	G	A	0.296371	0.0372935	1.91E-15	91.5217712	-0.0168343	0.067866	0.804094
European	rs116363527	T	C	-0.399579	0.0308305	2.05E-38	243.4231968	0.00573599	0.0546243	0.916369
European	rs116415754	T	C	-0.286518	0.0317616	1.87E-19	117.9284524	0.0512289	0.0743924	0.491055
European	rs12649106	T	C	-0.0936049	0.0124557	5.69E-14	81.84216381	-0.00180623	0.0198429	0.927471
European	rs144423926	A	C	0.444212	0.0488134	9.02E-20	120.0108932	-0.0299697	0.065731	0.64843
European	rs148935601	T	G	0.408853	0.042469	6.14E-22	134.310262	0.110435	0.0757597	0.144922
European	rs151287934	T	C	0.293484	0.0462741	2.26E-10	58.29223855	0.095405	0.0960692	0.320668
European	rs191462603	T	C	0.42587	0.0467444	8.19E-20	120.2858633	0.0157578	0.0613937	0.797435
European	rs36028675	C	T	0.182312	0.0144351	1.45E-36	231.1565446	-0.0021219	0.0224675	0.924757
European	rs505986	A	G	-0.0772975	0.0122345	2.65E-10	57.84664551	0.0293275	0.0196726	0.136019
European	rs55960593	T	C	-0.182379	0.012231	2.79E-50	322.2119517	0.0289523	0.0202446	0.152681
European	rs62351210	G	A	0.346526	0.0376388	3.37E-20	122.83379	-0.0371167	0.0773811	0.631469
European	rs62352866	A	G	0.153868	0.0121683	1.19E-36	231.7146512	-0.02946	0.01923	0.125526
European	rs72691475	A	G	0.282003	0.0481381	4.68E-09	49.73353549	0.0457446	0.0616977	0.458432
European	rs72971304	T	C	0.34466	0.038047	1.32E-19	118.9214506	-0.0444568	0.0897944	0.620533
European	rs76465512	A	G	-0.319234	0.0408975	5.92E-15	88.29655078	0.105241	0.103329	0.308439

Supplementary Table 8. Continued

Population	SNP	EA	OA	SNP-ETFDH				SNP-HER2- BC		
				beta	SE	P-value	F	beta	SE	P-value
European	rs77019759	A	G	-0.167177	0.0245614	1.00E-11	67.13737647	0.0118837	0.0362881	0.743303
European	rs77499728	C	T	-0.285343	0.0375961	3.21E-14	83.47713505	-0.0225129	0.0453203	0.619364
European	rs78628731	A	G	0.500249	0.0421429	1.69E-32	204.1941765	0.0149864	0.0454294	0.741488

SNP, single nucleotide polymorphisms; EA, effect allele; OA, other allele; SE, standard error; F, F-statistics.

Supplementary Table 9. Characteristics of ETFDH variants associated with ER-positive breast cancer (ER+ BC)

Population	SNP	EA	OA	SNP-ETFDH				SNP-ER+ BC		
				beta	SE	P-value	F	beta	SE	P-value
European	rs10517688	T	C	-0.0819387	0.0135608	1.52E-09	52.90825329	-0.0015	0.0468	0.9749
European	rs114481283	A	C	0.353578	0.0475341	1.02E-13	80.18206953	0.3683	0.213	0.0837703
European	rs115035697	T	C	0.239274	0.0392878	1.13E-09	53.75205699	-0.0074	0.1609	0.9631
European	rs11559290	T	C	0.329702	0.0178501	3.56E-76	494.4038596	-0.0744	0.0626	0.2344
European	rs115877139	G	A	0.296371	0.0372935	1.91E-15	91.5217712	-0.0112	0.1491	0.94
European	rs116363527	T	C	-0.399579	0.0308305	2.05E-38	243.4231968	0.2474	0.1074	0.0213098
European	rs116415754	T	C	-0.286518	0.0317616	1.87E-19	117.9284524	-0.049	0.124	0.692599
European	rs12649106	T	C	-0.0936049	0.0124557	5.69E-14	81.84216381	0.0274	0.0452	0.5439
European	rs144423926	A	C	0.444212	0.0488134	9.02E-20	120.0108932	0.301	0.2284	0.1874
European	rs148935601	T	G	0.408853	0.042469	6.14E-22	134.310262	0.0879	0.1753	0.6161
European	rs151287934	T	C	0.293484	0.0462741	2.26E-10	58.29223855	0.2179	0.2409	0.3656
European	rs191462603	T	C	0.42587	0.0467444	8.19E-20	120.2858633	-0.1276	0.1621	0.4313
European	rs1928260	T	A	0.149194	0.0224429	2.98E-11	64.04148696	-0.0831	0.0798	0.2982
European	rs36028675	C	T	0.182312	0.0144351	1.45E-36	231.1565446	-0.0169	0.0493	0.731301
European	rs505986	A	G	-0.0772975	0.0122345	2.65E-10	57.84664551	0.0208	0.0314	0.5066
European	rs55960593	T	C	-0.182379	0.012231	2.79E-50	322.2119517	0.0166	0.0314	0.5962
European	rs62351210	G	A	0.346526	0.0376388	3.37E-20	122.83379	-0.399	0.1618	0.0136499
European	rs62352866	A	G	0.153868	0.0121683	1.19E-36	231.7146512	-0.0088	0.0355	0.8054
European	rs6856561	T	A	0.491892	0.0130512	0	2058.544529	-0.0568	0.0468	0.2247
European	rs72691475	A	G	0.282003	0.0481381	4.68E-09	49.73353549	-0.2169	0.176	0.2179
European	rs72695442	A	G	0.306461	0.0374766	2.90E-16	96.90571081	0.1206	0.1419	0.3954

Supplementary Table 9. Continued

Population	SNP	EA	OA	SNP-ETFDH				SNP-ER+ BC		
				beta	SE	P-value	F	beta	SE	P-value
European	rs72971304	T	C	0.34466	0.038047	1.32E-19	118.9214506	0.0315	0.1425	0.8251
European	rs76465512	A	G	-0.319234	0.0408975	5.92E-15	88.29655078	0.0209	0.115	0.8558
European	rs77019759	A	G	-0.167177	0.0245614	1.00E-11	67.13737647	0.1615	0.0748	0.03092
European	rs77499728	C	T	-0.285343	0.0375961	3.21E-14	83.47713505	0.2365	0.1403	0.0919306
European	rs77797348	A	G	-0.13207	0.0221415	2.45E-09	51.56002488	-0.127	0.0771	0.0993711
European	rs78628731	A	G	0.500249	0.0421429	1.69E-32	204.1941765	-0.1228	0.1448	0.3963

SNP, single nucleotide polymorphisms; EA, effect allele; OA, other allele; SE, standard error; F, F-statistics.

Supplementary Table 10. Characteristics of ETFDH variants associated with ER-negative breast cancer (ER- BC)

Population	SNP	EA	OA	SNP-ETFDH				SNP-ER- BC		
				beta	SE	P-value	F	beta	SE	P-value
European	rs10517688	T	C	-0.0819387	0.0135608	1.52E-09	52.90825329	0.02	0.0363	0.582101
European	rs114481283	A	C	0.353578	0.0475341	1.02E-13	80.18206953	0.0649	0.1793	0.7172
European	rs115035697	T	C	0.239274	0.0392878	1.13E-09	53.75205699	0.0193	0.1237	0.8761
European	rs11559290	T	C	0.329702	0.0178501	3.56E-76	494.4038596	-0.0109	0.046	0.8127
European	rs115877139	G	A	0.296371	0.0372935	1.91E-15	91.5217712	0.0832	0.1152	0.47
European	rs116363527	T	C	-0.399579	0.0308305	2.05E-38	243.4231968	0.0952	0.0832	0.2524
European	rs116415754	T	C	-0.286518	0.0317616	1.87E-19	117.9284524	0.0319	0.0981	0.744801
European	rs12649106	T	C	-0.0936049	0.0124557	5.69E-14	81.84216381	-0.0138	0.0351	0.6937
European	rs144423926	A	C	0.444212	0.0488134	9.02E-20	120.0108932	-0.058	0.2278	0.7991
European	rs148935601	T	G	0.408853	0.042469	6.14E-22	134.310262	0.0526	0.1294	0.6844
European	rs151287934	T	C	0.293484	0.0462741	2.26E-10	58.29223855	0.142	0.1769	0.4222
European	rs191462603	T	C	0.42587	0.0467444	8.19E-20	120.2858633	-0.0181	0.1486	0.903
European	rs1928260	T	A	0.149194	0.0224429	2.98E-11	64.04148696	-0.1182	0.0637	0.0636107
European	rs36028675	C	T	0.182312	0.0144351	1.45E-36	231.1565446	0.042	0.0381	0.2703
European	rs505986	A	G	-0.0772975	0.0122345	2.65E-10	57.84664551	-0.009	0.0281	0.747901
European	rs55960593	T	C	-0.182379	0.012231	2.79E-50	322.2119517	0.0739	0.0281	0.00852707
European	rs62351210	G	A	0.346526	0.0376388	3.37E-20	122.83379	0.0479	0.1073	0.655101
European	rs62352866	A	G	0.153868	0.0121683	1.19E-36	231.7146512	-0.0378	0.0307	0.2184
European	rs6856561	T	A	0.491892	0.0130512	0	2058.544529	-4.00E-04	0.0349	0.9908
European	rs72691475	A	G	0.282003	0.0481381	4.68E-09	49.73353549	0.0094	0.1644	0.9544
European	rs72695442	A	G	0.306461	0.0374766	2.90E-16	96.90571081	0.0745	0.1201	0.5351

Supplementary Table 10. Continued

Population	SNP	EA	OA	SNP-ETFDH				SNP-ER- BC		
				beta	SE	P-value	F	beta	SE	P-value
European	rs72971304	T	C	0.34466	0.038047	1.32E-19	118.9214506	0.0734	0.1022	0.4721
European	rs76465512	A	G	-0.319234	0.0408975	5.92E-15	88.29655078	0.0597	0.0964	0.535601
European	rs77019759	A	G	-0.167177	0.0245614	1.00E-11	67.13737647	-0.0139	0.0639	0.8278
European	rs77499728	C	T	-0.285343	0.0375961	3.21E-14	83.47713505	0.2786	0.1251	0.02594
European	rs77797348	A	G	-0.13207	0.0221415	2.45E-09	51.56002488	-0.0204	0.0603	0.7356
European	rs78628731	A	G	0.500249	0.0421429	1.69E-32	204.1941765	0.0894	0.1505	0.5523

SNP, single nucleotide polymorphisms; EA, effect allele; OA, other allele; SE, standard error; F, F-statistics.

Supplementary Table 11. Characteristics of GPD1L variants associated with type 2 diabetes (T2D)

Population	SNP	EA	OA	SNP-GPD1L				SNP-T2D		
				beta	SE	P-value	F	beta	SE	P-value
European	rs10865840	G	A	-0.666001	0.0114235	0	7601.342688	-0.00116696	0.00814764	0.886112
European	rs11129513	T	C	-0.100187	0.0137003	2.62E-13	119.5923691	0.0186331	0.0100167	0.062855
European	rs111365533	T	C	-0.535343	0.0454482	5.00E-32	310.2916889	0.0306016	0.0324727	0.345998
European	rs111623195	A	G	0.4356	0.0506771	8.28E-18	165.2304992	0.106487	0.0805203	0.186008
European	rs111841306	C	T	-0.392946	0.0450771	2.85E-18	169.9385364	-0.031366	0.0286258	0.2732
European	rs114083031	G	A	-0.401236	0.0491085	3.07E-16	149.2879303	0.00332206	0.0365108	0.927502
European	rs114653385	A	G	-0.451928	0.0408985	2.19E-28	273.0623622	0.00606743	0.0189051	0.748256
European	rs116514552	C	T	-0.328589	0.0411791	1.47E-15	142.3937745	-0.0262383	0.0273345	0.337107
European	rs116789724	G	A	0.204892	0.0264428	9.30E-15	134.2687243	-0.018279	0.016704	0.27383
European	rs1451522	G	A	-0.214017	0.0384322	2.57E-08	69.34979211	0.0440799	0.047526	0.353673
European	rs147700828	C	T	0.436373	0.0396048	3.12E-28	271.4932402	0.0351203	0.0262007	0.180104
European	rs149650211	T	C	0.239087	0.0397479	1.80E-09	80.91399166	-0.0671218	0.0336478	0.0460606
European	rs2342367	A	G	0.181651	0.0279106	7.60E-11	94.72734051	0.0243884	0.023167	0.292468
European	rs35104436	A	G	0.0752044	0.0129533	6.41E-09	75.38110355	-0.00266335	0.0100961	0.791934
European	rs4269110	C	T	-0.0814789	0.0138069	3.61E-09	77.88151534	-0.00432617	0.0091269	0.635498
European	rs4334675	A	G	0.158384	0.018226	3.62E-18	168.8791547	-0.0424467	0.0162047	0.00880826
European	rs4398469	C	T	0.134141	0.0236981	1.51E-08	71.65266478	-0.0180171	0.0142649	0.206575
European	rs4955138	A	G	0.206689	0.0154429	7.49E-41	400.6060184	-0.00533928	0.0102166	0.601247
European	rs5025421	T	C	-0.0971275	0.0137723	1.76E-12	111.227474	-0.0124773	0.0100347	0.213713
European	rs56728060	C	A	0.110084	0.01494	1.73E-13	121.41873	0.00694913	0.0100658	0.489959
European	rs61519740	G	T	-0.221136	0.0304043	3.51E-13	118.3011128	0.0146065	0.0175277	0.404656

Supplementary Table 11. Continued

Population	SNP	EA	OA	SNP-GPD1L				SNP-T2D		
				beta	SE	P-value	F	beta	SE	P-value
European	rs62243257	C	T	0.279115	0.0224025	1.25E-35	347.1469356	-0.0024173	0.0143474	0.866203
European	rs62245378	G	A	0.292644	0.0328581	5.28E-19	177.3911478	-0.00411645	0.021477	0.848003
European	rs74367560	A	G	0.415327	0.0429807	4.33E-22	208.8202084	-0.0804943	0.0467793	0.0853022
European	rs74770257	G	A	0.196536	0.030807	1.78E-10	91.01736486	0.0253482	0.0362271	0.484113
European	rs75919845	C	A	-1.05793	0.0250308	0	3994.88585	0.00110466	0.0187816	0.953099
European	rs7611893	C	T	0.124924	0.0121961	1.27E-24	234.6318672	-0.00627372	0.00817468	0.44281
European	rs7634668	T	G	0.322186	0.0171727	1.56E-78	787.182383	0.00116501	0.0107303	0.913542
European	rs76585806	T	G	-0.285605	0.0170666	7.32E-63	626.2912546	0.00928149	0.0100838	0.357343
European	rs76925014	A	G	0.581075	0.0175271	5.14E-241	2458.000036	-0.0131074	0.0141085	0.352868
European	rs77050352	T	C	-0.556767	0.0258291	4.67E-103	1039.123947	-0.0110273	0.0179774	0.539612
European	rs77620813	A	C	-0.221285	0.0300536	1.80E-13	121.240897	0.00806806	0.028839	0.77966
European	rs77721561	C	T	-0.205112	0.033264	7.00E-10	85.03015061	0.00723594	0.0236281	0.75942
European	rs78138065	T	C	-0.232485	0.0214866	2.77E-27	261.8139137	0.0273192	0.0122225	0.0254074
European	rs79019422	G	A	-0.357583	0.0361901	5.05E-23	218.3305623	-0.0198976	0.0181911	0.274039
European	rs79694970	T	C	-0.406637	0.0371117	6.14E-28	268.4914423	0.0239375	0.0244452	0.327467
European	rs80019885	A	G	-0.12471	0.0210081	2.92E-09	78.80782942	0.0141092	0.0174979	0.420048
European	rs9822166	C	T	-0.16687	0.0276133	1.51E-09	81.66923236	0.00232853	0.0129158	0.856928
European	rs9842135	G	A	-0.101949	0.0131256	8.02E-15	134.917048	0.00365257	0.00855329	0.669353
European	rs9854375	C	A	-0.102243	0.0135775	5.06E-14	126.8137197	0.00548091	0.00953776	0.565525
European	rs9866851	T	G	-0.16568	0.0157516	7.11E-26	247.4164471	-0.019677	0.0119888	0.100739

SNP, single nucleotide polymorphisms; EA, effect allele; OA, other allele; SE, standard error; F, F-statistics.

Supplementary Table 12. Characteristics of GPD1L variants associated with Overall breast cancer (BC)

Population	SNP	EA	OA	SNP-GPD1L				SNP-Overall BC		
				beta	SE	P-value	F	beta	SE	P-value
European	rs10865840	G	A	-0.666001	0.0114235	0	7601.342688	-0.00709832	0.0123424	0.565212
European	rs11129513	T	C	-0.100187	0.0137003	2.62E-13	119.5923691	0.0168294	0.0152651	0.270255
European	rs111365533	T	C	-0.535343	0.0454482	5.00E-32	310.2916889	0.00573697	0.0487473	0.906315
European	rs111623195	A	G	0.4356	0.0506771	8.28E-18	165.2304992	0.224876	0.124822	0.0716143
European	rs111841306	C	T	-0.392946	0.0450771	2.85E-18	169.9385364	0.0240286	0.0428775	0.575207
European	rs114083031	G	A	-0.401236	0.0491085	3.07E-16	149.2879303	0.104395	0.0550925	0.0581059
European	rs114653385	A	G	-0.451928	0.0408985	2.19E-28	273.0623622	0.0216801	0.0287374	0.450597
European	rs116514552	C	T	-0.328589	0.0411791	1.47E-15	142.3937745	-0.0876056	0.041637	0.0353753
European	rs116789724	G	A	0.204892	0.0264428	9.30E-15	134.2687243	0.0180896	0.0251853	0.472597
European	rs1451522	G	A	-0.214017	0.0384322	2.57E-08	69.34979211	0.031858	0.0728251	0.661779
European	rs147700828	C	T	0.436373	0.0396048	3.12E-28	271.4932402	0.00297181	0.0401807	0.941041
European	rs149650211	T	C	0.239087	0.0397479	1.80E-09	80.91399166	-0.0435072	0.0506636	0.39048
European	rs2342367	A	G	0.181651	0.0279106	7.60E-11	94.72734051	0.0509556	0.0351659	0.147336
European	rs35104436	A	G	0.0752044	0.0129533	6.41E-09	75.38110355	0.00233441	0.0152869	0.878629
European	rs4269110	C	T	-0.0814789	0.0138069	3.61E-09	77.88151534	0.00728452	0.0138472	0.598844
European	rs4334675	A	G	0.158384	0.018226	3.62E-18	168.8791547	0.0412039	0.0243078	0.0900596
European	rs4398469	C	T	0.134141	0.0236981	1.51E-08	71.65266478	-0.0193212	0.0216559	0.37229
European	rs4955138	A	G	0.206689	0.0154429	7.49E-41	400.6060184	0.00597641	0.0155344	0.700443
European	rs5025421	T	C	-0.0971275	0.0137723	1.76E-12	111.227474	0.0252856	0.0152342	0.0969572
European	rs56728060	C	A	0.110084	0.01494	1.73E-13	121.41873	-0.0065726	0.0153575	0.66867
European	rs61519740	G	T	-0.221136	0.0304043	3.51E-13	118.3011128	0.00055324	0.0264678	0.983323

Supplementary Table 12. Continued

Population	SNP	EA	OA	SNP-GPD1L				SNP-Overall BC		
				beta	SE	P-value	F	beta	SE	P-value
European	rs62243257	C	T	0.279115	0.0224025	1.25E-35	347.1469356	-0.0281533	0.0218831	0.198258
European	rs62245378	G	A	0.292644	0.0328581	5.28E-19	177.3911478	-0.0511839	0.0328539	0.119251
European	rs74367560	A	G	0.415327	0.0429807	4.33E-22	208.8202084	0.0693897	0.073932	0.347956
European	rs74770257	G	A	0.196536	0.030807	1.78E-10	91.01736486	0.0838415	0.0547733	0.125844
European	rs75919845	C	A	-1.05793	0.0250308	0	3994.88585	-0.0211135	0.0285544	0.459656
European	rs7611893	C	T	0.124924	0.0121961	1.27E-24	234.6318672	-0.0104358	0.0123916	0.399697
European	rs7634668	T	G	0.322186	0.0171727	1.56E-78	787.182383	0.00789877	0.0163015	0.628002
European	rs76585806	T	G	-0.285605	0.0170666	7.32E-63	626.2912546	0.0179073	0.0152912	0.241562
European	rs76925014	A	G	0.581075	0.0175271	5.14E-241	2458.000036	-0.00422185	0.0215894	0.84496
European	rs77050352	T	C	-0.556767	0.0258291	4.67E-103	1039.123947	-0.0269497	0.0273178	0.323876
European	rs77620813	A	C	-0.221285	0.0300536	1.80E-13	121.240897	-0.04601	0.0440672	0.296446
European	rs77721561	C	T	-0.205112	0.033264	7.00E-10	85.03015061	-0.00521599	0.0359999	0.884799
European	rs78138065	T	C	-0.232485	0.0214866	2.77E-27	261.8139137	-0.0282915	0.0186119	0.128492
European	rs79019422	G	A	-0.357583	0.0361901	5.05E-23	218.3305623	-0.00734312	0.027701	0.790944
European	rs79694970	T	C	-0.406637	0.0371117	6.14E-28	268.4914423	-0.0143724	0.0369768	0.697508
European	rs80019885	A	G	-0.12471	0.0210081	2.92E-09	78.80782942	0.00302252	0.0267004	0.909871
European	rs9822166	C	T	-0.16687	0.0276133	1.51E-09	81.66923236	0.0157355	0.0196033	0.422151
European	rs9842135	G	A	-0.101949	0.0131256	8.02E-15	134.917048	0.00590229	0.0130194	0.650299
European	rs9854375	C	A	-0.102243	0.0135775	5.06E-14	126.8137197	0.00225742	0.0144682	0.876012
European	rs9866851	T	G	-0.16568	0.0157516	7.11E-26	247.4164471	0.0334524	0.0182184	0.06633

SNP, single nucleotide polymorphisms; EA, effect allele; OA, other allele; SE, standard error; F, F-statistics.

Supplementary Table 13. Characteristics of GPD1L variants associated with HER2-positive breast cancer (HER2+ BC)

Population	SNP	EA	OA	SNP-GPD1L				SNP-HER2+ BC		
				beta	SE	P-value	F	beta	SE	P-value
European	rs10865840	G	A	-0.666001	0.0114235	0	7601.342688	-0.0179426	0.0152399	0.239058
European	rs11129513	T	C	-0.100187	0.0137003	2.62E-13	119.5923691	0.0323676	0.0188547	0.086036
European	rs111365533	T	C	-0.535343	0.0454482	5.00E-32	310.2916889	0.0218537	0.0600483	0.715906
European	rs111623195	A	G	0.4356	0.0506771	8.28E-18	165.2304992	0.361722	0.153953	0.0187953
European	rs111841306	C	T	-0.392946	0.0450771	2.85E-18	169.9385364	0.0239752	0.0530456	0.651288
European	rs114083031	G	A	-0.401236	0.0491085	3.07E-16	149.2879303	0.0660622	0.0682604	0.333146
European	rs114653385	A	G	-0.451928	0.0408985	2.19E-28	273.0623622	-0.00944813	0.0355249	0.790271
European	rs116514552	C	T	-0.328589	0.0411791	1.47E-15	142.3937745	-0.0871088	0.051335	0.0897222
European	rs116789724	G	A	0.204892	0.0264428	9.30E-15	134.2687243	0.0498637	0.0310776	0.108606
European	rs1451522	G	A	-0.214017	0.0384322	2.57E-08	69.34979211	-0.0168485	0.0890046	0.849858
European	rs147700828	C	T	0.436373	0.0396048	3.12E-28	271.4932402	-0.00843911	0.0495977	0.864891
European	rs149650211	T	C	0.239087	0.0397479	1.80E-09	80.91399166	-0.0160808	0.0624695	0.796856
European	rs2342367	A	G	0.181651	0.0279106	7.60E-11	94.72734051	0.0635766	0.0436063	0.144849
European	rs35104436	A	G	0.0752044	0.0129533	6.41E-09	75.38110355	0.017994	0.018875	0.340426
European	rs4269110	C	T	-0.0814789	0.0138069	3.61E-09	77.88151534	-0.00619091	0.0171051	0.717401
European	rs4334675	A	G	0.158384	0.018226	3.62E-18	168.8791547	0.04846	0.0300595	0.106933
European	rs4398469	C	T	0.134141	0.0236981	1.51E-08	71.65266478	-0.0283711	0.0267642	0.289127
European	rs4955138	A	G	0.206689	0.0154429	7.49E-41	400.6060184	0.0130126	0.0191729	0.497328
European	rs5025421	T	C	-0.0971275	0.0137723	1.76E-12	111.227474	0.00525916	0.0187789	0.779433
European	rs56728060	C	A	0.110084	0.01494	1.73E-13	121.41873	-0.00330253	0.0189817	0.861877
European	rs61519740	G	T	-0.221136	0.0304043	3.51E-13	118.3011128	0.00445376	0.0327109	0.891699

Supplementary Table 13.　Continued

Population	SNP	EA	OA	SNP-GPD1L				SNP-HER2+ BC		
				beta	SE	P-value	F	beta	SE	P-value
European	rs62243257	C	T	0.279115	0.0224025	1.25E-35	347.1469356	-0.0311479	0.0270158	0.24893
European	rs62245378	G	A	0.292644	0.0328581	5.28E-19	177.3911478	-0.0302088	0.0405055	0.455791
European	rs74367560	A	G	0.415327	0.0429807	4.33E-22	208.8202084	0.0348886	0.0912229	0.702124
European	rs74770257	G	A	0.196536	0.030807	1.78E-10	91.01736486	0.0899108	0.0678647	0.18522
European	rs75919845	C	A	-1.05793	0.0250308	0	3994.88585	-0.0456479	0.0353516	0.196616
European	rs7611893	C	T	0.124924	0.0121961	1.27E-24	234.6318672	-0.00367186	0.0153098	0.810456
European	rs7634668	T	G	0.322186	0.0171727	1.56E-78	787.182383	0.015871	0.0201777	0.431539
European	rs76585806	T	G	-0.285605	0.0170666	7.32E-63	626.2912546	0.0275319	0.0189002	0.1452
European	rs76925014	A	G	0.581075	0.0175271	5.14E-241	2458.000036	-0.0156253	0.0267072	0.558509
European	rs77050352	T	C	-0.556767	0.0258291	4.67E-103	1039.123947	-0.0586597	0.033782	0.0824898
European	rs77620813	A	C	-0.221285	0.0300536	1.80E-13	121.240897	-0.0348962	0.0542762	0.520264
European	rs77721561	C	T	-0.205112	0.033264	7.00E-10	85.03015061	0.0332473	0.0443493	0.453454
European	rs78138065	T	C	-0.232485	0.0214866	2.77E-27	261.8139137	-0.0515802	0.0230041	0.0249471
European	rs79019422	G	A	-0.357583	0.0361901	5.05E-23	218.3305623	-0.0123793	0.0343116	0.718257
European	rs79694970	T	C	-0.406637	0.0371117	6.14E-28	268.4914423	-0.0487823	0.0457775	0.286587
European	rs80019885	A	G	-0.12471	0.0210081	2.92E-09	78.80782942	0.00622046	0.0329089	0.850077
European	rs9822166	C	T	-0.16687	0.0276133	1.51E-09	81.66923236	0.0153367	0.0242172	0.52654
European	rs9842135	G	A	-0.101949	0.0131256	8.02E-15	134.917048	0.00256162	0.0160813	0.873438
European	rs9854375	C	A	-0.102243	0.0135775	5.06E-14	126.8137197	0.0106789	0.0178764	0.550258
European	rs9866851	T	G	-0.16568	0.0157516	7.11E-26	247.4164471	0.0254841	0.0224867	0.257089

SNP, single nucleotide polymorphisms; EA, effect allele; OA, other allele; SE, standard error; F, F-statistics.

Supplementary Table 14. Characteristics of GPD1L variants associated with HER2-negative breast cancer (HER2- BC)

Population	SNP	EA	OA	SNP-GPD1L				SNP-HER2- BC		
				beta	SE	P-value	F	beta	SE	P-value
European	rs10865840	G	A	-0.666001	0.0114235	0	7601.342688	0.014545	0.0191256	0.446957
European	rs11129513	T	C	-0.100187	0.0137003	2.62E-13	119.5923691	-0.00466294	0.0236405	0.843637
European	rs111365533	T	C	-0.535343	0.0454482	5.00E-32	310.2916889	-0.0334634	0.07564	0.658197
European	rs111623195	A	G	0.4356	0.0506771	8.28E-18	165.2304992	0.00175349	0.198637	0.992957
European	rs111841306	C	T	-0.392946	0.0450771	2.85E-18	169.9385364	0.0222603	0.0663646	0.737306
European	rs114083031	G	A	-0.401236	0.0491085	3.07E-16	149.2879303	0.165836	0.0856054	0.0527193
European	rs114653385	A	G	-0.451928	0.0408985	2.19E-28	273.0623622	0.0831322	0.0445986	0.0623204
European	rs116514552	C	T	-0.328589	0.0411791	1.47E-15	142.3937745	-0.0912492	0.0640567	0.1543
European	rs116789724	G	A	0.204892	0.0264428	9.30E-15	134.2687243	-0.0323816	0.039121	0.407824
European	rs1451522	G	A	-0.214017	0.0384322	2.57E-08	69.34979211	0.145809	0.113171	0.19761
European	rs147700828	C	T	0.436373	0.0396048	3.12E-28	271.4932402	0.0155416	0.06215	0.802537
European	rs149650211	T	C	0.239087	0.0397479	1.80E-09	80.91399166	-0.0998677	0.0783004	0.202152
European	rs2342367	A	G	0.181651	0.0279106	7.60E-11	94.72734051	0.0454981	0.0543885	0.402852
European	rs35104436	A	G	0.0752044	0.0129533	6.41E-09	75.38110355	-0.0146448	0.0237052	0.536713
European	rs4269110	C	T	-0.0814789	0.0138069	3.61E-09	77.88151534	0.0301693	0.0214421	0.159425
European	rs4334675	A	G	0.158384	0.018226	3.62E-18	168.8791547	0.0361562	0.0377238	0.337838
European	rs4398469	C	T	0.134141	0.0236981	1.51E-08	71.65266478	-0.00685253	0.0335473	0.838147
European	rs4955138	A	G	0.206689	0.0154429	7.49E-41	400.6060184	-0.0039183	0.0240363	0.870506
European	rs5025421	T	C	-0.0971275	0.0137723	1.76E-12	111.227474	0.0626465	0.0238043	0.00849533
European	rs56728060	C	A	0.110084	0.01494	1.73E-13	121.41873	-0.00868861	0.0237686	0.7147
European	rs61519740	G	T	-0.221136	0.0304043	3.51E-13	118.3011128	-0.00556698	0.0409301	0.891812

Supplementary Table 14. Continued

Population	SNP	EA	OA	SNP-GPD1L				SNP-HER2- BC		
				beta	SE	P-value	F	beta	SE	P-value
European	rs62243257	C	T	0.279115	0.0224025	1.25E-35	347.1469356	-0.0250908	0.0338488	0.458536
European	rs62245378	G	A	0.292644	0.0328581	5.28E-19	177.3911478	-0.0897221	0.0508939	0.077913
European	rs74367560	A	G	0.415327	0.0429807	4.33E-22	208.8202084	0.112594	0.115303	0.328813
European	rs74770257	G	A	0.196536	0.030807	1.78E-10	91.01736486	0.0817338	0.0845994	0.33398
European	rs75919845	C	A	-1.05793	0.0250308	0	3994.88585	0.0284408	0.0442247	0.520161
European	rs7611893	C	T	0.124924	0.0121961	1.27E-24	234.6318672	-0.0241211	0.0191838	0.208621
European	rs7634668	T	G	0.322186	0.0171727	1.56E-78	787.182383	-0.00848505	0.0252115	0.736453
European	rs76585806	T	G	-0.285605	0.0170666	7.32E-63	626.2912546	0.00122361	0.0237065	0.958835
European	rs76925014	A	G	0.581075	0.0175271	5.14E-241	2458.000036	0.012676	0.0333585	0.70395
European	rs77050352	T	C	-0.556767	0.0258291	4.67E-103	1039.123947	0.0281941	0.0422533	0.504604
European	rs77620813	A	C	-0.221285	0.0300536	1.80E-13	121.240897	-0.0723289	0.0679552	0.287165
European	rs77721561	C	T	-0.205112	0.033264	7.00E-10	85.03015061	-0.0668708	0.0558606	0.231267
European	rs78138065	T	C	-0.232485	0.0214866	2.77E-27	261.8139137	0.00762073	0.02879	0.79124
European	rs79019422	G	A	-0.357583	0.0361901	5.05E-23	218.3305623	0.0010553	0.0427898	0.980324
European	rs79694970	T	C	-0.406637	0.0371117	6.14E-28	268.4914423	0.043005	0.056947	0.450142
European	rs80019885	A	G	-0.12471	0.0210081	2.92E-09	78.80782942	-0.00653427	0.0412091	0.874013
European	rs9822166	C	T	-0.16687	0.0276133	1.51E-09	81.66923236	0.0210439	0.0304081	0.488906
European	rs9842135	G	A	-0.101949	0.0131256	8.02E-15	134.917048	0.00979025	0.0201605	0.62724
European	rs9854375	C	A	-0.102243	0.0135775	5.06E-14	126.8137197	-0.00847154	0.0224054	0.705354
European	rs9866851	T	G	-0.16568	0.0157516	7.11E-26	247.4164471	0.0472525	0.0280807	0.0924251

SNP, single nucleotide polymorphisms; EA, effect allele; OA, other allele; SE, standard error; F, F-statistics.

Supplementary Table 15. Characteristics of GPD1L variants associated with ER-positive breast cancer (ER+ BC)

Population	SNP	EA	OA	SNP-GPD1L				SNP-ER+ BC		
				beta	SE	P-value	F	beta	SE	P-value
European	rs10865840	G	A	-0.666001	0.0114235	0	7601.342688	-0.0095	0.0436	0.8279
European	rs11129513	T	C	-0.100187	0.0137003	2.62E-13	119.5923691	0.0846	0.052	0.1035
European	rs111365533	T	C	-0.535343	0.0454482	5.00E-32	310.2916889	0.1512	0.1687	0.3701
European	rs111623195	A	G	0.4356	0.0506771	8.28E-18	165.2304992	0.0743	0.208	0.721
European	rs111841306	C	T	-0.392946	0.0450771	2.85E-18	169.9385364	0.2711	0.1594	0.089
European	rs114083031	G	A	-0.401236	0.0491085	3.07E-16	149.2879303	-0.1194	0.2027	0.5559
European	rs114653385	A	G	-0.451928	0.0408985	2.19E-28	273.0623622	-0.0853	0.1442	0.5544
European	rs116514552	C	T	-0.328589	0.0411791	1.47E-15	142.3937745	0.0664	0.1445	0.6461
European	rs116789724	G	A	0.204892	0.0264428	9.30E-15	134.2687243	0.0349	0.1018	0.731601
European	rs1451522	G	A	-0.214017	0.0384322	2.57E-08	69.34979211	0.1582	0.1608	0.3254
European	rs147700828	C	T	0.436373	0.0396048	3.12E-28	271.4932402	-0.0972	0.1498	0.5164
European	rs149650211	T	C	0.239087	0.0397479	1.80E-09	80.91399166	-0.0069	0.1469	0.9625
European	rs2342367	A	G	0.181651	0.0279106	7.60E-11	94.72734051	0.0484	0.0802	0.5461
European	rs35104436	A	G	0.0752044	0.0129533	6.41E-09	75.38110355	0.0131	0.0343	0.702
European	rs4269110	C	T	-0.0814789	0.0138069	3.61E-09	77.88151534	0.0063	0.0346	0.8561
European	rs4303904	T	A	-0.115338	0.021077	4.44E-08	66.96749487	0.0021	0.0714	0.9762
European	rs4334675	A	G	0.158384	0.018226	3.62E-18	168.8791547	-0.0448	0.0499	0.3701
European	rs4398469	C	T	0.134141	0.0236981	1.51E-08	71.65266478	-0.0484	0.0686	0.48
European	rs4955138	A	G	0.206689	0.0154429	7.49E-41	400.6060184	0.0155	0.0533	0.771999
European	rs5025421	T	C	-0.0971275	0.0137723	1.76E-12	111.227474	-0.0283	0.0426	0.5068
European	rs56376585	T	A	-0.226852	0.0182328	1.55E-35	346.1935447	0.001	0.0503	0.9839

Supplementary Table 15. Continued

Population	SNP	EA	OA	SNP-GPD1L				SNP-ER+ BC		
				beta	SE	P-value	F	beta	SE	P-value
European	rs56728060	C	A	0.110084	0.01494	1.73E-13	121.41873	0.0029	0.0556	0.9585
European	rs61519740	G	T	-0.221136	0.0304043	3.51E-13	118.3011128	-0.1134	0.1263	0.3694
European	rs62243257	C	T	0.279115	0.0224025	1.25E-35	347.1469356	-0.0348	0.0761	0.6477
European	rs62245378	G	A	0.292644	0.0328581	5.28E-19	177.3911478	-0.0146	0.1124	0.8968
European	rs74367560	A	G	0.415327	0.0429807	4.33E-22	208.8202084	-0.1234	0.2101	0.5569
European	rs74770257	G	A	0.196536	0.030807	1.78E-10	91.01736486	-0.0709	0.1133	0.5313
European	rs75919845	C	A	-1.05793	0.0250308	0	3994.88585	-0.0202	0.0895	0.8213
European	rs7611893	C	T	0.124924	0.0121961	1.27E-24	234.6318672	-0.0098	0.0417	0.8147
European	rs7623335	T	C	-0.10166	0.0154229	4.35E-11	97.1641235	-0.0062	0.0342	0.8564
European	rs7634668	T	G	0.322186	0.0171727	1.56E-78	787.182383	-0.0146	0.0577	0.8006
European	rs76585806	T	G	-0.285605	0.0170666	7.32E-63	626.2912546	0.0247	0.0589	0.674899
European	rs76925014	A	G	0.581075	0.0175271	5.14E-241	2458.000036	-0.0561	0.0615	0.3616
European	rs77050352	T	C	-0.556767	0.0258291	4.67E-103	1039.123947	-8.00E-04	0.069	0.9911
European	rs77620813	A	C	-0.221285	0.0300536	1.80E-13	121.240897	-0.141	0.1123	0.209
European	rs77721561	C	T	-0.205112	0.033264	7.00E-10	85.03015061	0.0203	0.1045	0.8457
European	rs78138065	T	C	-0.232485	0.0214866	2.77E-27	261.8139137	0.0475	0.0509	0.3508
European	rs79019422	G	A	-0.357583	0.0361901	5.05E-23	218.3305623	-0.341	0.1464	0.01986
European	rs79694970	T	C	-0.406637	0.0371117	6.14E-28	268.4914423	-0.0735	0.1407	0.6017
European	rs79923561	T	G	0.206814	0.015028	4.32E-43	423.53817	0.0224	0.041	0.5841
European	rs80019885	A	G	-0.12471	0.0210081	2.92E-09	78.80782942	0.039	0.0756	0.6058
European	rs9816495	C	G	-0.0784365	0.0135887	7.82E-09	74.51118595	0.0492	0.0348	0.1575

Supplementary Table 15. Continued

Population	SNP	EA	OA	SNP-GPD1L				SNP-ER+ BC		
				beta	SE	P-value	F	beta	SE	P-value
European	rs9822166	C	T	-0.16687	0.0276133	1.51E-09	81.66923236	-0.1966	0.0943	0.0371501
European	rs9842135	G	A	-0.101949	0.0131256	8.02E-15	134.917048	0.0041	0.0439	0.9249
European	rs9854375	C	A	-0.102243	0.0135775	5.06E-14	126.8137197	0.0186	0.0356	0.601399
European	rs9866851	T	G	-0.16568	0.0157516	7.11E-26	247.4164471	-0.0111	0.0402	0.782299

SNP, single nucleotide polymorphisms; EA, effect allele; OA, other allele; SE, standard error; F, F-statistics.

Supplementary Table 16. Characteristics of GPD1L variants associated with ER-negative breast cancer (ER- BC)

Population	SNP	EA	OA	SNP-GPD1L				SNP-ER- BC		
				beta	SE	P-value	F	beta	SE	P-value
European	rs10865840	G	A	-0.666001	0.0114235	0	7601.342688	-0.0012	0.0334	0.971
European	rs11129513	T	C	-0.100187	0.0137003	2.62E-13	119.5923691	0	0.0391	0.9997
European	rs111365533	T	C	-0.535343	0.0454482	5.00E-32	310.2916889	0.052	0.1247	0.676799
European	rs111623195	A	G	0.4356	0.0506771	8.28E-18	165.2304992	-0.0282	0.1719	0.8697
European	rs111841306	C	T	-0.392946	0.0450771	2.85E-18	169.9385364	0.079	0.1201	0.511
European	rs114083031	G	A	-0.401236	0.0491085	3.07E-16	149.2879303	-0.0486	0.1484	0.743401
European	rs114653385	A	G	-0.451928	0.0408985	2.19E-28	273.0623622	-0.0836	0.1362	0.5394
European	rs116514552	C	T	-0.328589	0.0411791	1.47E-15	142.3937745	0.0389	0.1254	0.756201
European	rs116789724	G	A	0.204892	0.0264428	9.30E-15	134.2687243	0.0742	0.0734	0.3121
European	rs1451522	G	A	-0.214017	0.0384322	2.57E-08	69.34979211	-0.1634	0.1163	0.1601
European	rs147700828	C	T	0.436373	0.0396048	3.12E-28	271.4932402	0.1393	0.1215	0.2515
European	rs149650211	T	C	0.239087	0.0397479	1.80E-09	80.91399166	-0.0929	0.1178	0.4302
European	rs2342367	A	G	0.181651	0.0279106	7.60E-11	94.72734051	0.033	0.0686	0.6304
European	rs35104436	A	G	0.0752044	0.0129533	6.41E-09	75.38110355	0.0289	0.0307	0.3473
European	rs4269110	C	T	-0.0814789	0.0138069	3.61E-09	77.88151534	-0.0309	0.0312	0.3229
European	rs4303904	T	A	-0.115338	0.021077	4.44E-08	66.96749487	0.0317	0.0516	0.5392
European	rs4334675	A	G	0.158384	0.018226	3.62E-18	168.8791547	-0.0686	0.0431	0.1115
European	rs4398469	C	T	0.134141	0.0236981	1.51E-08	71.65266478	-0.0558	0.0597	0.3502
European	rs4955138	A	G	0.206689	0.0154429	7.49E-41	400.6060184	0.0388	0.0415	0.349
European	rs5025421	T	C	-0.0971275	0.0137723	1.76E-12	111.227474	-0.0243	0.0353	0.490399
European	rs56376585	T	A	-0.226852	0.0182328	1.55E-35	346.1935447	0.0287	0.0433	0.5067

Supplementary Table 16.   Continued

Population	SNP	EA	OA	SNP-GPD1L			F	SNP-ER- BC		
				beta	SE	P-value	F	beta	SE	P-value
European	rs56728060	C	A	0.110084	0.01494	1.73E-13	121.41873	0.0228	0.0413	0.5807
European	rs61519740	G	T	-0.221136	0.0304043	3.51E-13	118.3011128	-0.2181	0.1313	0.096859
European	rs62243257	C	T	0.279115	0.0224025	1.25E-35	347.1469356	-0.0392	0.0608	0.519501
European	rs62245378	G	A	0.292644	0.0328581	5.28E-19	177.3911478	-0.0016	0.0894	0.9854
European	rs74367560	A	G	0.415327	0.0429807	4.33E-22	208.8202084	0.1975	0.1718	0.2503
European	rs74770257	G	A	0.196536	0.030807	1.78E-10	91.01736486	-0.0176	0.0801	0.8262
European	rs75919845	C	A	-1.05793	0.0250308	0	3994.88585	0.006	0.0714	0.9328
European	rs7611893	C	T	0.124924	0.0121961	1.27E-24	234.6318672	-0.0173	0.0314	0.5817
European	rs7623335	T	C	-0.10166	0.0154229	4.35E-11	97.1641235	-0.0243	0.0305	0.4256
European	rs7634668	T	G	0.322186	0.0171727	1.56E-78	787.182383	0.067	0.0477	0.1601
European	rs76585806	T	G	-0.285605	0.0170666	7.32E-63	626.2912546	0.0593	0.0439	0.1763
European	rs76925014	A	G	0.581075	0.0175271	5.14E-241	2458.000036	0.0178	0.0465	0.702301
European	rs77050352	T	C	-0.556767	0.0258291	4.67E-103	1039.123947	0.0093	0.0628	0.8819
European	rs77620813	A	C	-0.221285	0.0300536	1.80E-13	121.240897	0.0878	0.0863	0.309
European	rs77721561	C	T	-0.205112	0.033264	7.00E-10	85.03015061	0.1672	0.0798	0.0361402
European	rs78138065	T	C	-0.232485	0.0214866	2.77E-27	261.8139137	3.00E-04	0.0486	0.9956
European	rs79019422	G	A	-0.357583	0.0361901	5.05E-23	218.3305623	0.0942	0.1215	0.4382
European	rs79694970	T	C	-0.406637	0.0371117	6.14E-28	268.4914423	-0.0192	0.1257	0.8788
European	rs79923561	T	G	0.206814	0.015028	4.32E-43	423.53817	0.0612	0.0362	0.0905691
European	rs80019885	A	G	-0.12471	0.0210081	2.92E-09	78.80782942	0.0018	0.0561	0.9741
European	rs9816495	C	G	-0.0784365	0.0135887	7.82E-09	74.51118595	0.0484	0.0307	0.1144

Supplementary Table 16. Continued

Population	SNP	EA	OA	SNP-GPD1L				SNP-ER- BC		
				beta	SE	P-value	F	beta	SE	P-value
European	rs9822166	C	T	-0.16687	0.0276133	1.51E-09	81.66923236	-0.1039	0.0809	0.1986
European	rs9842135	G	A	-0.101949	0.0131256	8.02E-15	134.917048	-0.0141	0.0327	0.6675
European	rs9854375	C	A	-0.102243	0.0135775	5.06E-14	126.8137197	0.012	0.0311	0.7002
European	rs9866851	T	G	-0.16568	0.0157516	7.11E-26	247.4164471	-0.0267	0.0352	0.4486

SNP, single nucleotide polymorphisms; EA, effect allele; OA, other allele; SE, standard error; F, F-statistics.

Supplementary Table 17. Sensitivity analysis, heterogeneity, and pleiotropy, investigating MR assumption violation

Exposure - Outcome	Heterogeneity tests						Tests for directional horizontal pleiotropy		
	Inverse variance weighted			MR Egger			Egger_intercept	SE	P-value
	Q	Q_df	Q_pval	Q	Q_df	Q_pval			
PRKAB1 - T2D	118.3290815	39	6.36E-10	117.3574272	38	4.98E-10	0.003490182	0.006222368	0.578152306
PRKAB1 - Overall BC	73.57503541	41	0.001338933	73.35617049	40	0.001011664	0.002339487	0.006772062	0.73155763
PRKAB1 - HER2+ BC	149.1523378	11	2.22E-26	82.25158451	10	1.81E-13	-0.061444496	0.021544659	0.017192236
PRKAB1 - HER2- BC	297.5475554	244	0.010836253	297.1433009	243	0.010066325	-0.003710025	0.006452518	0.565841375
ETFDH - T2D	34.7219765	24	0.072631124	34.63043842	23	0.056575289	0.001742673	0.007067734	0.807430332
ETFDH - Overall BC	31.32846014	23	0.114935184	29.39910552	22	0.133815122	-0.012486946	0.010392163	0.242304671
ETFDH - HER2+ BC	26.71464393	23	0.268303685	25.28207587	22	0.28377662	-0.013289711	0.011902904	0.276250344
ETFDH - HER2- BC	19.0104358	23	0.70061874	18.32058368	22	0.686820719	-0.01155544	0.013912607	0.415137805
ETFDH - ER+ BC	30.00554231	26	0.267381481	30.00550666	25	0.224082881	-0.000142152	0.026084521	0.99569507
ETFDH - ER- BC	22.55061926	26	0.658227314	21.79146752	25	0.647755404	-0.017043822	0.019561524	0.391884615
GPD1L - T2D	45.3519729	40	0.258680861	43.48505778	39	0.286221722	-0.004680175	0.003616908	0.203286052
GPD1L - Overall BC	43.32846119	40	0.331270709	40.01592554	39	0.424865595	-0.009467575	0.005269175	0.080117743
GPD1L - HER2+ BC	42.41294603	40	0.367383391	41.39068645	39	0.366721081	-0.006496975	0.006619873	0.33242767
GPD1L - HER2- BC	36.29978096	40	0.6375726	33.63484017	39	0.712717341	-0.013157191	0.008059709	0.110631012
GPD1L - ER+ BC	29.58224742	45	0.963055612	29.18250473	44	0.958126555	-0.008477582	0.013408547	0.530494027
GPD1L - ER- BC	37.36249329	45	0.783579384	37.2207151	44	0.755353311	0.0041796	0.011100179	0.708329322
SE, standard error; Q, Cochran's Q statistic; T2D, type 2 diabetes; BC, breast cancer.									

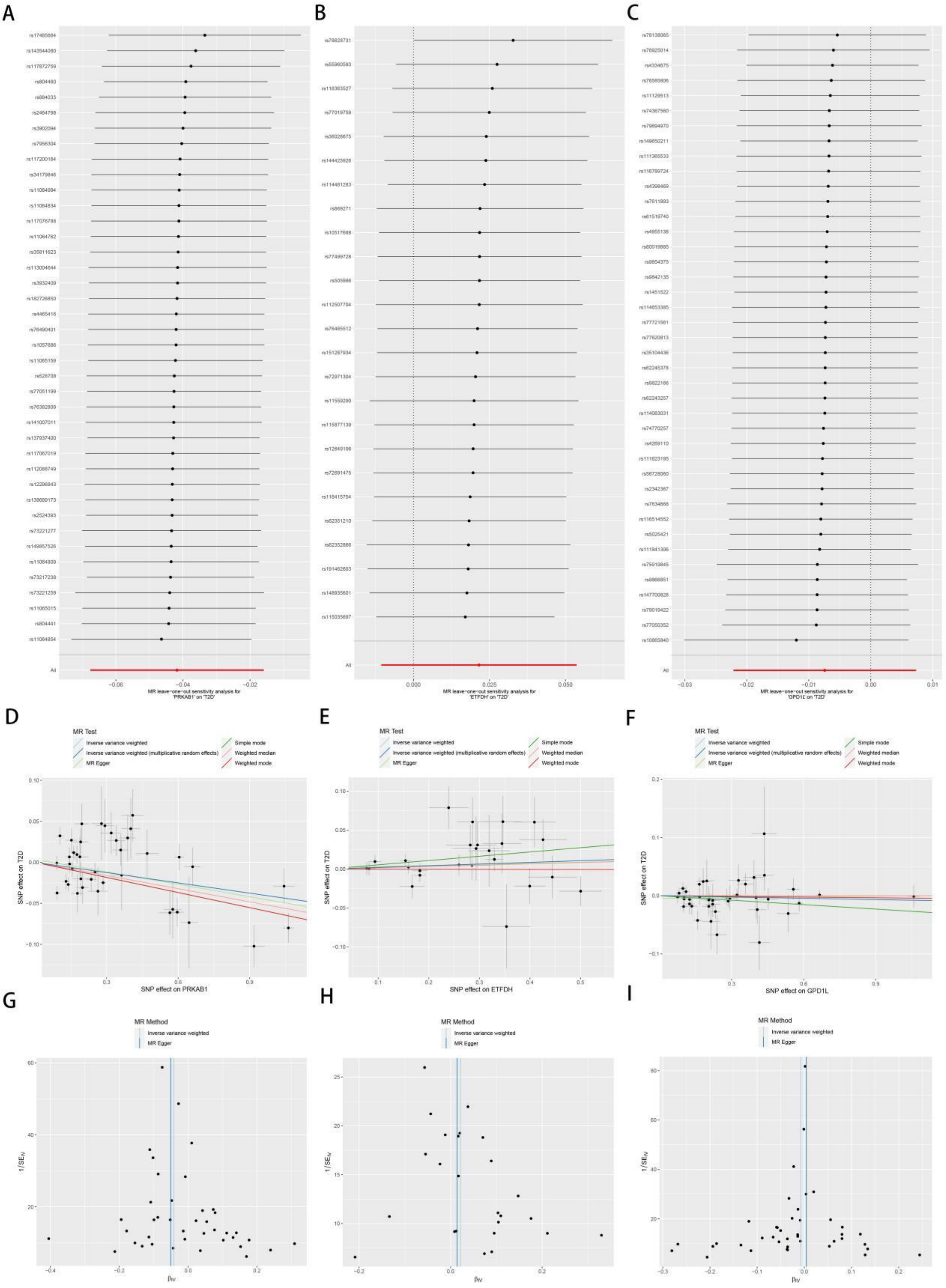

Figure S1 Sensitivity analysis of metformin targets on type 2 diabetes (T2D). Leave-one-out analysis of (A)PRKAB1,(B) ETFDH and(C) GPD1L on T2D. Scatter plots for the association between (D)PRKAB1,(E) ETFDH ,(F) GPD1L and T2D. Funnel plots for the association between (G)PRKAB1,(H) ETFDH ,(I) GPD1L and T2D.

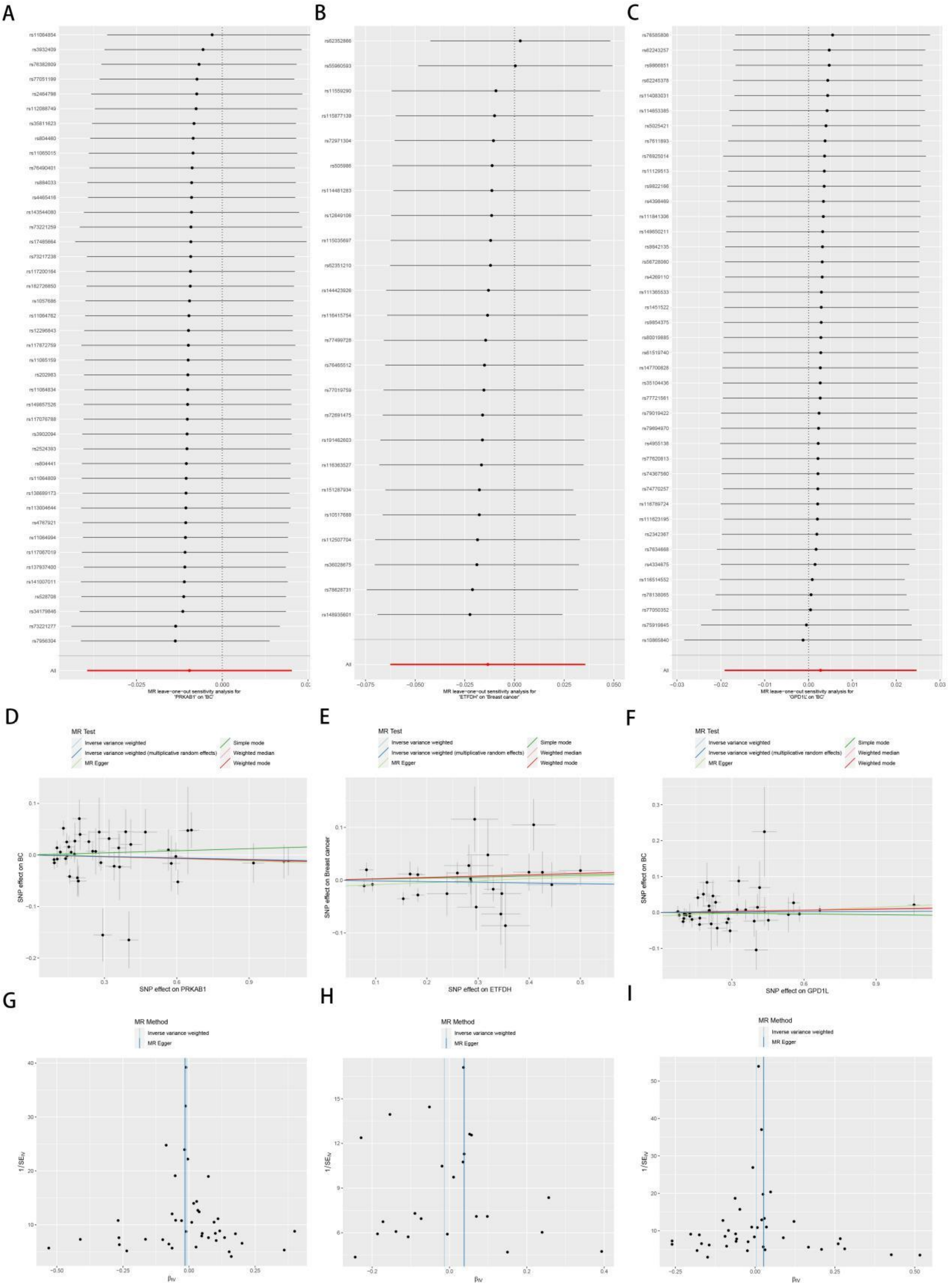

Figure S2 Sensitivity analysis of metformin targets on overall breast cancer (BC).
Leave-one-out analysis of (A)PRKAB1,(B) ETFDH and(C) GPD1L on overall BC. Scatter plots for the association between (D)PRKAB1,(E) ETFDH ,(F) GPD1L and overall BC. Funnel plots for the association between (G)PRKAB1,(H) ETFDH ,(I) GPD1L and overall BC.

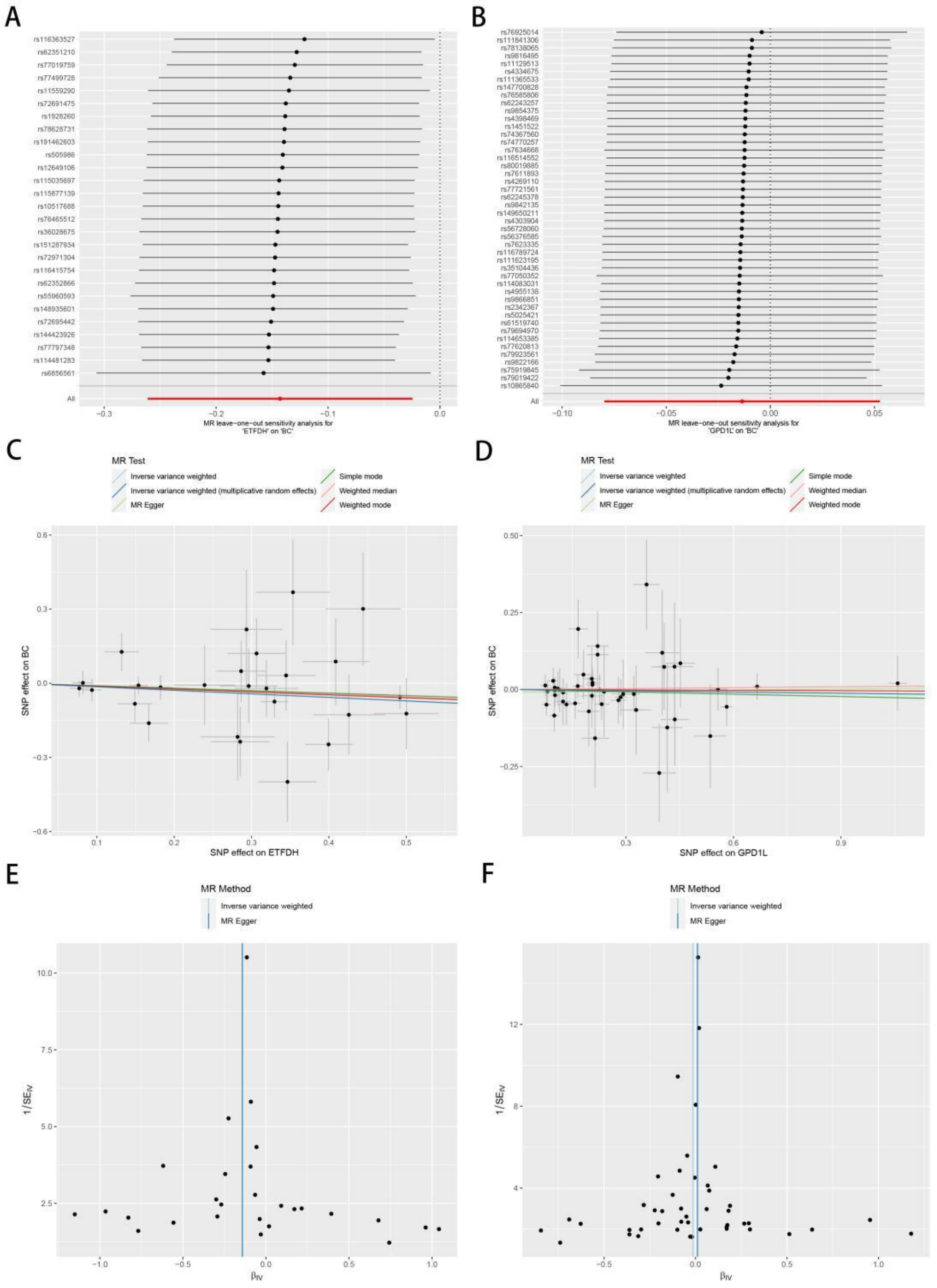

Figure S3 Sensitivity analysis of metformin targets on ER-positive breast cancer (BC). Leave-one-out analysis of (A) ETFDH and (B) GPD1L on ER-positive BC. Scatter plots for the association between (C) ETFDH and (D) GPD1L on ER-positive BC. Funnel plots for the association between (E) ETFDH and (F) GPD1L on ER-positive BC.

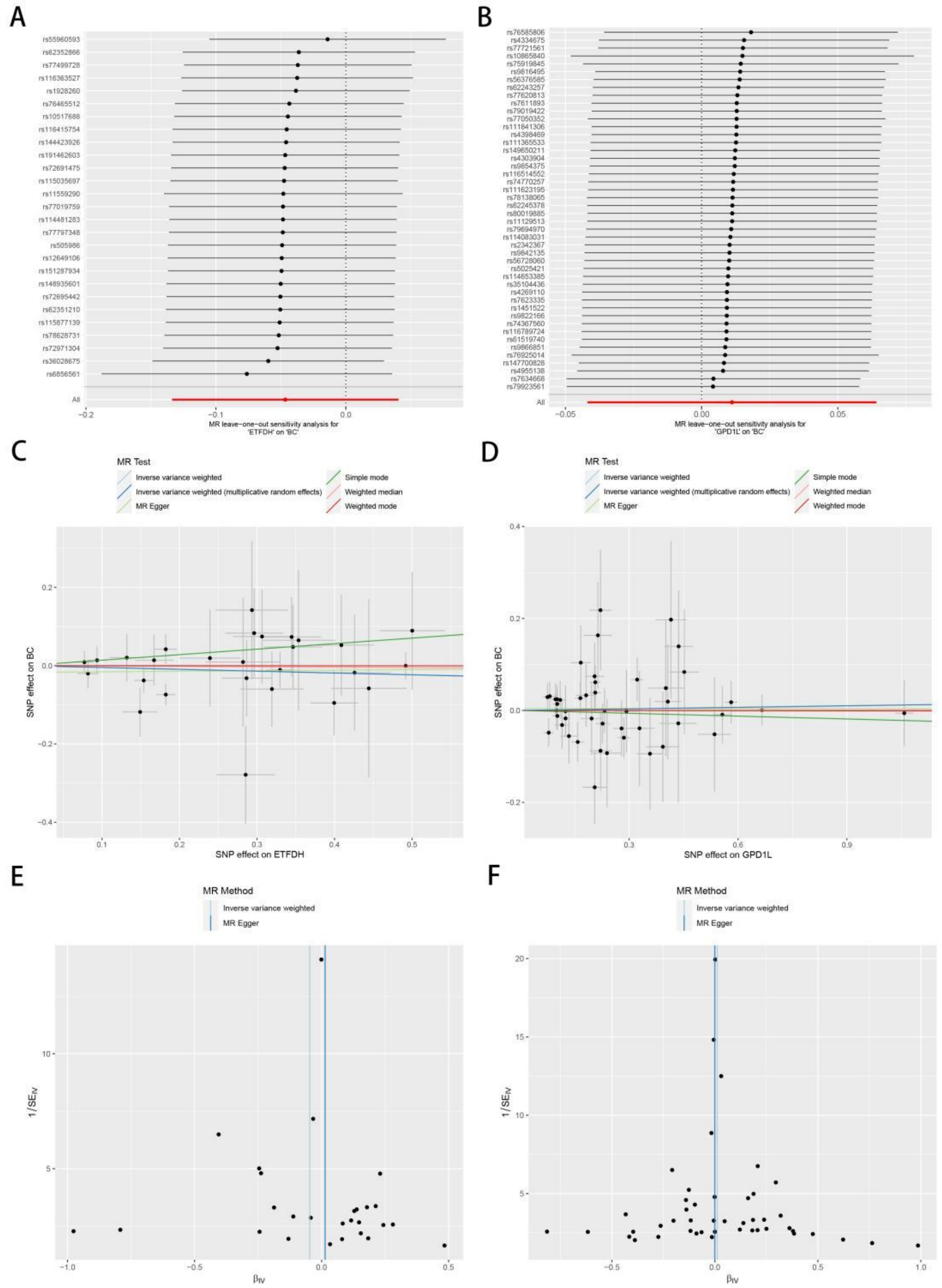

Figure S4 Sensitivity analysis of metformin targets on ER-negative breast cancer (BC). Leave-one-out analysis of (A) ETFDH and (B) GPD1L on ER-negative BC. Scatter plots for the association between (C) ETFDH and (D) GPD1L on ER-negative BC. Funnel plots for the association between (E) ETFDH and (F) GPD1L on ER-negative BC.

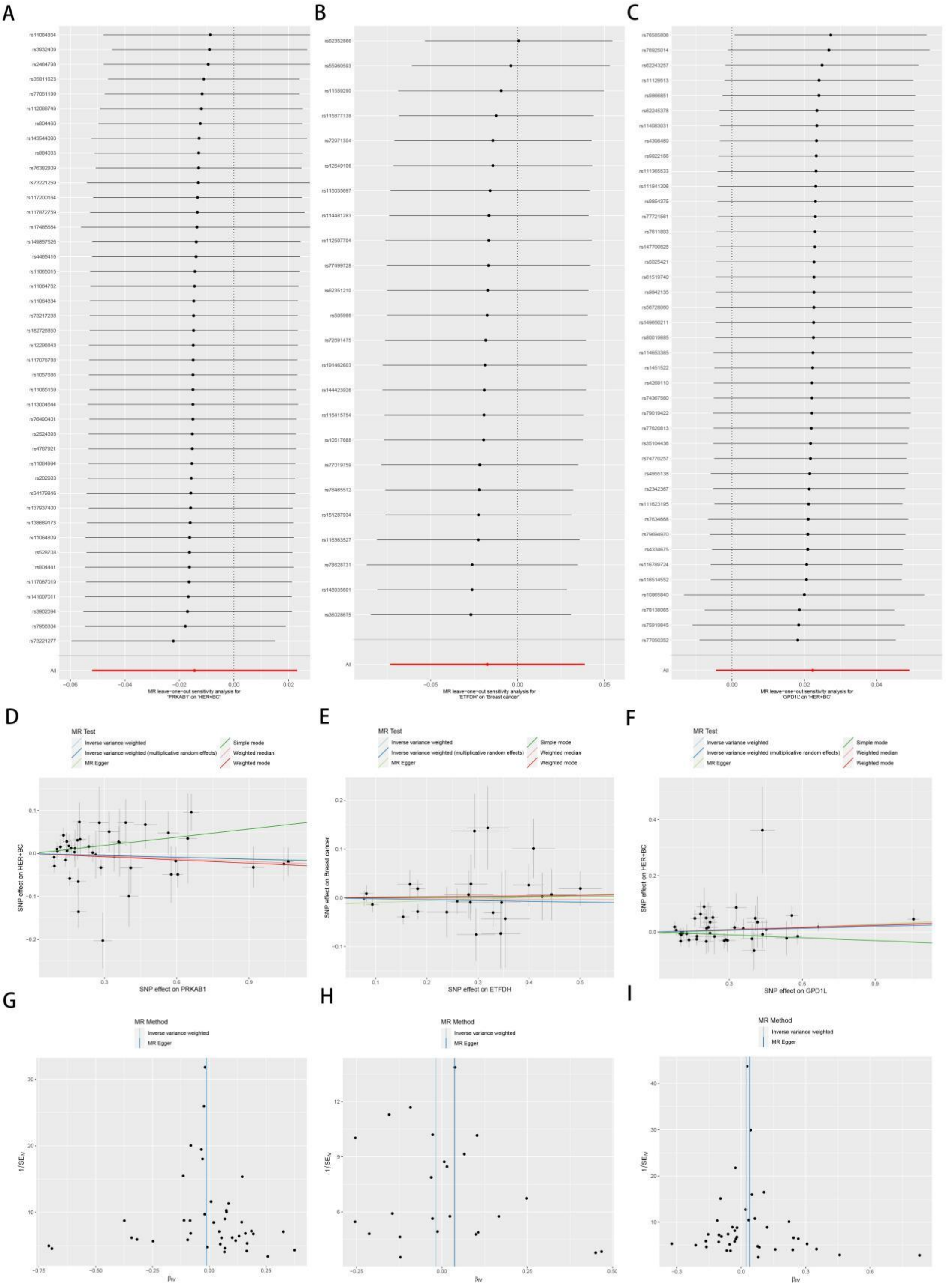

Figure S5 Sensitivity analysis of metformin targets on HER2-positive breast cancer (BC). Leave-one-out analysis of (A)PRKAB1,(B) ETFDH and(C) GPD1L on HER2-positive BC. Scatter plots for the association between (D)PRKAB1,(E) ETFDH ,(F) GPD1L and HER2-positive BC. Funnel plots for the association between (G)PRKAB1,(H) ETFDH ,(I) GPD1L and HER2-positive BC.

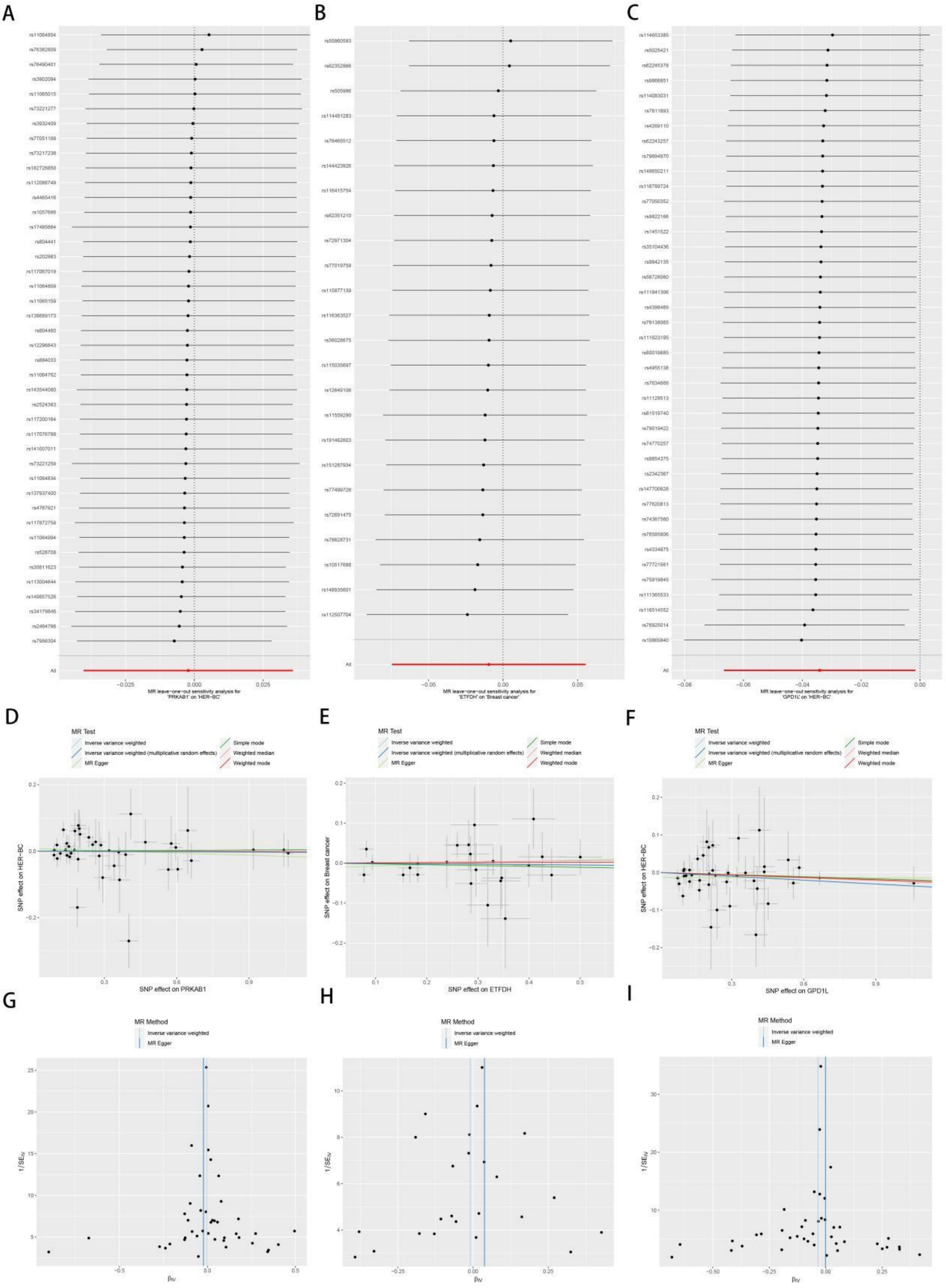

Figure S6 Sensitivity analysis of metformin targets on HER2-negative breast cancer (BC). Leave-one-out analysis of (A)PRKAB1,(B) ETFDH and(C) GPD1L on HER2-negative BC. Scatter plots for the association between (D)PRKAB1,(E) ETFDH ,(F) GPD1L and HER2-negative BC. Funnel plots for the association between (G)PRKAB1,(H) ETFDH ,(I) GPD1L and HER2-negative BC.
